# Promoting mental health in the workplace: web software development and validation *

**DOI:** 10.1590/1518-8345.7181.4353

**Published:** 2024-09-23

**Authors:** Evelin Daiane Gabriel Pinhatti, Regina Celia Bueno Rezende Machado, Rosangela Aparecida Pimenta, André Estevam Jaques, Maria do Carmo Fernandez Lourenço Haddad

**Affiliations:** ^1^ Universidade Estadual de Londrina, Londrina, PR, Brasil.; ^2^ Universidade Anhanguera, Unidade Catuaí, Londrina, PR, Brasil.; ^3^ Universidade Estadual de Maringá, Maringá, PR, Brasil.

**Keywords:** Mental Health, Occupational Health, Workplace, Information Technology, Software, Nursing

## Abstract

**Objective:**

to develop and validate the content and technical aspects of a web software program for promoting mental health in the workplace.

**Method:**

applied methodological study and technological development, carried out in three stages: 1) Umbrella review development; 2) Web software development; 3) Content and technical validation carried out by 14 judges. The data was submitted to descriptive statistical analysis and calculation of the content validity index.

**Results:**

based on the guidelines’ recommendations, information was defined and extracted in order to develop the web software consisting of the following dimensions: mental health education, support among coworkers, promotion strategies and mental health self-assessment. For the technical development, the objectives, general functions and technological infrastructure were defined. After development and functional testing, the version was made available for content and technical validation by judges. The overall content validity index was 0.98 and for the technical aspects it was 0.97.

**Conclusion:**

the agreement between the judges in relation to the content and technical aspects, as well as the suggestions incorporated, demonstrated the potential for using web software to promote mental health in the workplace.

## Highlights

**(1)** Use of technology to provide information on mental health.

**(2)** It allows information to be disseminated in a flexible and less stigmatizing way.

**(3)** Useful for preventing, tracking and dealing with mental health problems.

**(4)** Contributes to managers and occupational nurses in the workers’ assessment.

**(5)** Makes it possible to develop institutional strategies to promote mental health.

## Introduction

 Workers’ mental illness has become a major public health problem worldwide and the main cause of absence from work due to illness and disability ^(^ 1 ^)^ . It is estimated that 15% of adults suffer from some kind of mental disorder during their working lives. Depression and anxiety have emerged as the main causes of incapacity at work, and each year both contribute to approximately 12 billion lost working days, costing the global economy almost a trillion dollars ^(^ 2 ^)^ . 

 In Brazil, between 2007 and 2022 there were 17,681 notifications of work-related mental disorders ^(^ 3 ^)^ . Faced with this scenario, legislative proposals have been discussed to grant tax incentives to legal entities that implement mental health programs within the workplace, demonstrating an important advance for workers’ health ^(^ 4 ^)^ . 

 As far as causality is concerned, work alone does not act as the main cause of mental illness. However, it can be a contributory causal element, in other words, it can cause a latent disorder or aggravate a pre-existing illness ^(^ 5 ^)^ . 

 The World Health Organization (WHO) and the International Labor Organization (ILO) have called for concrete action to address the working population’s mental health concerns. Through the guidelines published, it is emphasized that the approach to mental health must be broad and comprehensive, and that it is important for work environments to adopt a scenario conducive to change, with a commitment to combating stigma and discrimination, coordinating multisectoral approaches ^(^ 2 ^)^ . 

 Impaired mental health affects people all over the world, impacting on individual quality of life and economically for employers ^(^ 6 ^)^ . Evidence has shown that implementing interventions in the workplace can promote mental health by improving the individual’s psychological aspect ^(^ 1 ^)^ . Based on the assumption that some mental health problems are preventable, there are sufficient arguments for employers to invest in preventive solutions for workers’ mental health ^(^ 6 ^)^ . 

 In recent years, digital health technologies for desktop and mobile devices have begun to be used to increase access to information. These technologies can increase the accessibility of mental health strategies. They can also reduce the challenges of a health professional shortage by minimizing the barriers arising from the need to meet face-to-face ^(^ 6 ^-^ 7 ^)^ . In this way, they can improve the gaps in the implementation of strategies and mimic the problems associated with mental health ^(^ 8 ^)^ . 

 Furthermore, technological innovations can increase awareness and adherence to prevention, education and support measures for individuals affected by mental health conditions, especially in the workplace, where traditional approaches tend to have low acceptance ^(^ 6 ^-^ 7 ^,^ 9 ^)^ . The use of technology can also be considered useful and advantageous for user access, as some people would not seek help due to fear of being stigmatized ^(^ 9 ^)^ . 

 The use of technologies to prevent or improve mental health problems can include actions based on software programs, accessed through computers, tablets, smartphones, audiovisual equipment, robots and other devices. The devices have resources for collecting, storing and retrieving information, as well as guiding users in carrying out therapeutic activities ^(^ 10 ^)^ . 

 In Brazil, the literature shows that the software production has been aimed at organizing health services ^(^ 11 ^-^ 12 ^)^ , managing nursing care ^(^ 13 ^)^ , teaching nursing ^(^ 14 ^-^ 15 ^)^ and patient self-care ^(^ 16 ^)^ . 

In view of the above, and given that there is a gap in the national scientific knowledge produced on the development of technologies to promote workers’ mental health, this study’s research question was: Does the web software developed to promote mental health in the workplace have content and technical validity?

In order to answer this research question, the aim of this study was to develop and validate the content and technical aspects of a web-based software program for promoting mental health in the workplace.

## Method

### Study design

 This is an applied methodological and technological development study aimed at developing or refining methods for obtaining, organizing or analyzing data, with a focus on developing, validating and evaluating tools ^(^ 17 ^)^ . 

### Period

The study was carried out from January 2021 to December 2022.

### Study procedures

The study was conducted in three stages: 1) To support the web software development, an umbrella review study was carried out to identify guideline recommendations for the mental health promotion in the workplace; 2) Web software development; 3) Web software content and technical validation by judges.

In the first stage, a search was carried out for systematic reviews that identified guideline recommendations for promoting mental health in the workplace.

The search was carried out in five databases: American Psychological Association (PsycINFO), Cochrane Library, EMBASE National Library of Medicine National Institutes of Health (MEDLINE via PubMed) and Scopus. The descriptors “mental health”, “workplace”, “guidelines” and “systematic review” were used. The umbrella review protocol was registered on the Prospero platform CRD42023461845.

The results were grouped into three categories of recommendations: a) Primary prevention recommendations - protecting workers; b) Secondary prevention recommendations - promoting workers’ mental health; and c) Tertiary prevention recommendations - supporting, monitoring and rehabilitating workers on their return to work after leave.

 Based on the evaluation of the recommendations described in the guidelines, information was defined and extracted for the following contents to be inserted into the web software: mental health awareness, social support for coworkers, promotion strategies and mental health self-assessment. The self-assessment was based on the Depression, Anxiety and Stress Scale (DASS-21), which has already been translated and validated for Brazil ^(^ 18 ^)^ . 

 In the second stage, i.e. designing the web software, methodological steps based on software engineering were used ^(^ 19 ^)^ . This model allows web software to be developed from a series of evolutionary versions involving the communication, planning, modeling, construction, evaluation, functional testing and validation phases. It also allows for any necessary adaptations throughout the life of the software ^(^ 19 ^)^ . 

To develop the web software, contact was made with a professional with expertise in systems development, through systematic meetings, where planning was defined, including the web software’s main requirements and resources, as well as a preliminary schedule for its development.

 The web software was modeled using a flowchart with the interface screens. The layout visible to the user was outlined, defining the layout of the screens and access icons. The web software was developed based on the concepts of Responsive Web Design, which allows it to be accessed on a desktop, tablet or smartphone by changing the layout in relation to the size of the screens, making it easier to view. The architecture used was the Content Management System (CMS). This tool concentrates various functions aimed at facilitating the creation and editing of content. The programming language used was Personal Home Page (PHP), Hyper Text Markup Language (HTML5), Cascading Style Sheets (CSS3) and Javascript ^(^ 20 ^)^ . 

 Adobe Photoshop ^®^ , Javascript with the Vue.js framework, HTML5 and CSS3 were used to create the frontend of the web software, which comprises the interface visible to the user. For the backend, which involves processing and storing data, the MySQL database manager was used. A framework was used to manage the content, which is managed through access to the tool’s administrative panel, making it easier for researchers to update it. The framework was hosted on the Linux/Apache server. During the testing phase, the need for adjustments to the interface and functionality was verified by testing links and forms ^(^ 20 ^)^ . 

 The third stage consisted of content and technical aspects being validated by judges on the recommendation of six or more specialists ^(^ 21 ^)^ . The judges were selected through the Lattes Platform, by intentional non-probabilistic sampling, and who met the inclusion criteria. 

The following inclusion criteria were established for the content judge: minimum doctoral degree; author/supervisor of studies related to workers’ health and/or mental health; in teaching, teaches/has taught courses/lectures on workers’ health and/or mental health; participates/has participated in groups/projects related to workers’ health and/or mental health.

The following criteria were established for the technical judge: specialist degree; author/supervisor of studies related to software development; teaches/has taught courses/lectures on software development; participates/has participated in groups/projects related to software development.

A total of 22 judges were invited, through contact via an e-mail invitation letter. Of these, eight did not return the call, resulting in a sample of 14 judges, eight of whom were nurses and six software development specialists.

### Instruments used to collect the information

To collect the data, two separate forms were drawn up using the Google Forms tool. The form included the Informed Consent Form (ICF) and the e-mail address for accessing the web software, along with the items in the validation instrument. A 15-day deadline was set for evaluating and completing the instrument, and two more attempts were made to send it back with the same deadline for responses. Failure to complete the questionnaire was considered a refusal.

 The content validation tool consisted of 13 statements covering items on objectives, content, relevance and environment. The technical validation instrument was made up of 14 statements covering items on ergonomics, functionality, usability and environment. Each of the dimensions contained items assessed using a four-point Likert scale, distributed as not relevant, slightly relevant, relevant and highly relevant. At the end of each dimension, there was a space for the judges’ suggestions. The instruments were adapted from a validation study of a web-based software called *Aposentar-se com Saúde* (Retiring Healthy), which was adapted to questions about promoting mental health in the workplace ^(^ 22 ^)^ . 

### Data analysis

 The data obtained was analyzed using descriptive statistics, using absolute and relative frequency values, with the help of Microsoft Office Excel ^®^ and Statistical Package for the Social Sciences (SPSS) ^®^ version 25.0. To analyze technical and content validity, the Content Validity Index (CVI) was calculated, considering the proportion of relevant or highly relevant responses divided by the total number of responses for each item. The CVI of the instrument’s dimensions was obtained from the sum of the dimension’s CVIs, divided by the number of items. Finally, the overall CVI was obtained by adding the individual CVIs divided by the total number of items in the instrument. The minimum value for the CVI was 0.78 ^(^ 21 ^)^ . The suggestions sent in by the judges were organized and analyzed according to the instrument’s dimensions. 

### Ethical aspects

This study complies with Resolution No. 466, of December 12, 2012, and was previously approved by the institution’s Research Ethics Committee (CEP), according to Opinion No. 3.588.273.

## Results

**Figure 1 -:**
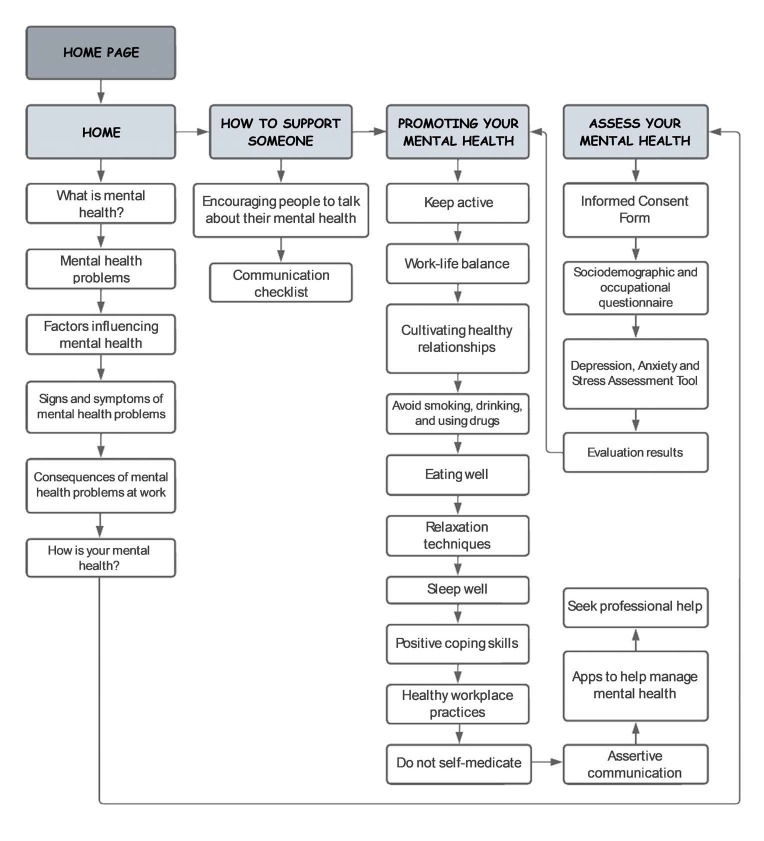
Web software flowchart for promoting mental health in the workplace. Londrina, PR, Brazil, 2023

 The web software was called *e-LeveMente* , it is hosted on a web server and can be accessed via the link https://www.elevemente.com . For the development, a meeting was held with the developers to define the objectives and general functions, where the technological infrastructure was drawn up with a flowchart, defining the content sequence, the media selection, the way the screens are integrated and their functions, as shown in Figure 1 . 

**Figure 2 -:**
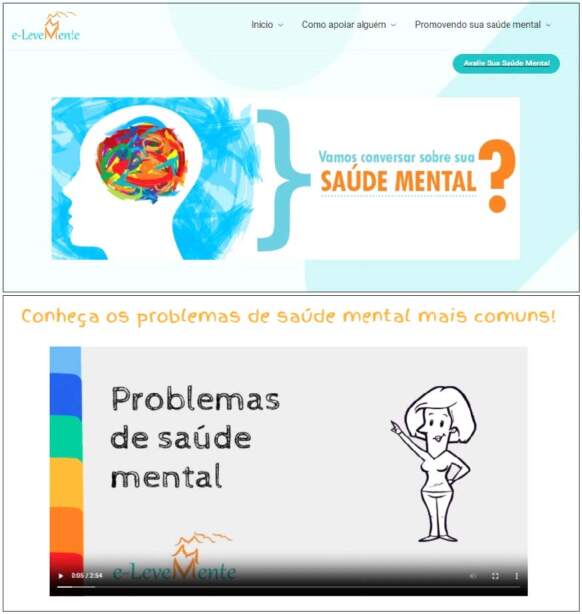
Graphic representation of the home screen and content of the web software for promoting mental health in the workplace. Londrina, PR, Brazil, 2023

 For the technical development, the home screen and presentation layout were defined ( Figure 2 ). The content was made available in the form of text, videos and images, in order to provide the user with an inviting and attractive environment. When accessing *e-LeveMente* , the top right corner of the screen displays the main sections and resources, which include: the home page, the how to support someone session, the promoting your mental health session and the assess your mental health icon ( Figure 2 ). 

The home page provides information on the mental health definition, common mental health problems, factors influencing mental health, signs and symptoms of mental health problems and the consequences of mental health problems at work.

 The session *how to support someone* , provides information on how to facilitate an initial conversation and approach the subject, what to ask, choosing the appropriate place, the importance of maintaining confidentiality and reassuring the person, how to encourage the person to talk and seek professional support, considered strategies for establishing effective communication. 

 In the *promoting your mental health* section, content is provided for self-care. Strategies include guidelines such as: the importance of staying active, establishing a work-life balance, cultivating healthy relationships, warnings about the risks of smoking, drinking and using drugs to reduce mental health-related discomfort, the importance of eating well, relaxation techniques, habits to improve sleep quality, positive coping skills, healthy workplace practices, warnings not to self-medicate in the event of mental health-related signs and symptoms, practices for assertive communication, suggestions for apps to help manage mental health and information on where to seek professional help. 

 In all sections, the *assess your mental health* icon is available in the top right-hand corner of the screen. By selecting *start evaluation* , the user is directed to a screen where they can view the ICF and, after reading and agreeing, they are directed to the screen containing the sociodemographic and occupational characterization questionnaire. The questions on the *web software interface* are compulsory and allow the user to move on to the next screen once they have completed it. 

On the next screen, the instrument for self-assessment and screening of mental health conditions is made available. Before starting the answers, the following information is given: “The questionnaire is an objective, reliable and clear measure for the user to understand how they are feeling. The result of the assessment does not indicate a conclusive diagnosis, but rather the starting point for stimulating self-care or seeking professional help”.

The questions were made available on three screens with seven questions, so that the user can enter an answer option for each question, allowing them to move from one screen to the next directly, using the forward or back buttons. At the end of the questionnaire, the software itself calculates the scores according to the scale’s recommendations, providing each individual’s score and generating a page with the results. The result is divided between the three factors: depression, anxiety and stress, and classified as normal, minimal, moderate, severe and very severe.

**Figure 3 -:**
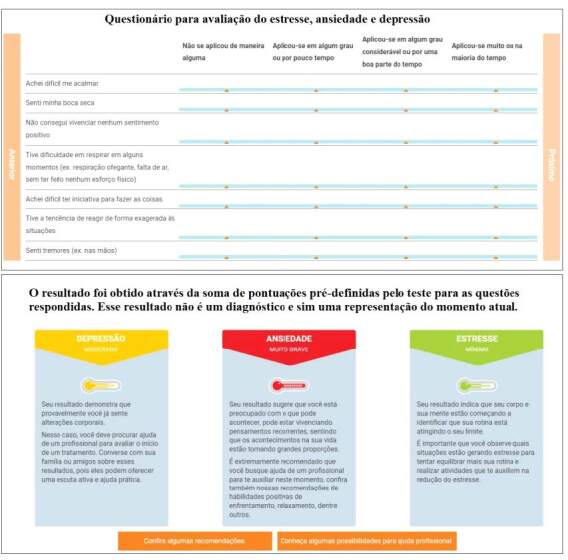
Graphic representation of the mental health self-assessment questionnaire and the results screen of the web software for promoting mental health in the workplace. Londrina, PR, Brazil, 2023

 According to the score, strategies for promoting mental health are indicated, as well as possibilities for professional help, which are accessed via the available link ( Figure 3 ). The answers to the questionnaires are stored in the *e-LeveMente* database and can be accessed via the administrative area and exported for future analysis. 

 It should be noted that during the *e-LeveMente* development process, tests were carried out to identify possible errors in aesthetics, content and links, with the aim of building a tool with adequate functionality. After the functional tests, the version was made available for content and technical validation by the judges. 

For content validation, eight content judges provided feedback. The mean education period was 25.4 years. Of these, 25% had a post-doctoral degree, 87.5% had experience in occupational health and 87.5% had experience in mental health.

 In the first content validation cycle, the overall CVI was 0.85. In relation to the dimensions in which the items were distributed, the one with the lowest CVI was the content domain, with a 0.79 CVI. Despite having an acceptable CVI, five of the items evaluated (2.2; 2.3; 2.5; 2.6 and 3.2) did not reach the minimum CVI adopted in this study. Therefore, as the judges made suggestions to improve the quality of the *e-LeveMente* content, these were evaluated for reformulation. 

 In relation to the judges’ considerations, in the Objective Dimension, it was suggested that information on mental health promotion be made available in an open format, considering that the user would only have access to it after completing the self-assessment questionnaire. This suggestion was complied with, and the section on the main menu *promoting your mental health* was created. 

 In the Content Dimension, changes to the font style and grammatical revision of the information were suggested. In the characterization questionnaire, in the gender item, it was suggested to include the option *prefer not to declare* . In relation to the mental health self-assessment questionnaire, it was recommended that the full reference be included, not just the authors’ names. 

In relation to mental health promotion strategies, it was recommended to replace the term “fostering relationships” with “cultivating healthy relationships” and to include the word “avoid” before: smoking, drinking and using drugs. In addition, it was suggested to include information on self-medication, sleep and assertive communication. For relaxation techniques, the suggestion was to include some free Android and iPhone Operating System (iOS) apps for relaxation and meditation.

For the Relevance Dimension, the format used to present the content on the mental health consequences at work was highlighted as excellent, as were the strategies to help workers talk about their mental health and the relevant guidelines in the communication checklist. It was also considered relevant, when assessing their mental health, to provide guidance on the results and strategies for promoting mental health. Some judges stressed that the mental health identification was being achieved, but that the strategies needed to be improved, as pointed out in the Content Dimension.

In the Environment Dimension, the judges pointed out that the soft colors and figures were appropriate and with excellent design. The material is easy to approach, with meaningful and relevant content for workers’ mental health, well-designed videos, clear and able to convey the necessary information and guidance. All that was recommended was to diversify the melodies in the videos and reduce the playback speed so that the user can assimilate the information more easily.

 It should be noted that the suggestions made by the judges were evaluated and incorporated into the new *e-LeveMente* version for validation. The appropriate version was then submitted for a new validation cycle. Seven judges gave feedback and the overall CVI was 0.98, as shown in Table 1 . 

In the technical validation, six judges gave feedback. The mean education period was 11.8 years, with degrees in Systems Analysis, Computer Science, Computer Engineering, Software Development Engineering and Computer Networking. Of these, 16.7% had a doctorate, 33.3% had a master’s degree and 50% were specialists.

**Table 1 -:** Content validity index and percentage agreement between the web software content validation judges for the mental health promotion in the workplace. Londrina, PR, Brazil, 2023

**Validation items**	**1° Percentage agreement**					**2° Percentage agreement**				
	1* %	**2** ^†^ **%**	**3** ^‡^ **%**	**4** ^§^ **%**	**CVI** ^||^	1* %	**2** ^†^ **%**	**3** ^‡^ **%**	**4** ^§^ **%**	**CVI** ^||^
**1. Objectives** 1.1 The web software’s objectives are consistent with the mental health promotion.	12.5	0.0	37.5	50.0	0.88	0.0	0.0	28.6	71.4	1.00
1.2 The web software makes it easier to understand the mental health promotion topic.	0.0	0.0	37.5	62.5	1.00	0.0	0.0	28.6	71.4	1.00
1.3 The proposed objectives are adequate to achieve them.	0.0	12.5	25.0	62.5	0.88	0.0	0.0	28.6	71.4	1.00
**CVI** ^||^ **- Objective Dimension**					**0.92**					**1.00**
**2. Content** 2.1 The web software’s contents correspondto its objectives.	0.0	12.5	37.5	50.0	0.88	0.0	0.0	28.6	71.4	1.00
2.2 The web software’s contents are sufficient to achieve the proposed objectives.	0.0	25.0	62.5	12.5	0.75	0.0	0.0	57.1	42.9	1.00
2.3 The web software’s contents precisely cover the scope of the topic.	0.0	37.5	50.0	12.5	0.63	0.0	0.0	42.9	57.1	1.00
2.4 The information presented is correct.	0.0	0.0	62.5	37.5	1.00	0.0	0.0	14.3	85.7	1.00
2.5 The wording is suitable for the health care workers’ different knowledge levels.	0.0	25.0	25.0	50.0	0.75	0.0	0.0	28.6	71.4	1.00
2.6 The contents make it easier to understand the different dimensions of mental health promotion.	0.0	25.0	37.5	37.5	0.75	0.0	0.0	42.9	57.1	1.00
**CVI** ^||^ **- Content Dimension**					**0.79**					**1.00**
**3. Relevance** 3.1 The contents address key aspects that should be explored in the mental health promotion.	0.0	12.5	25.0	62.5	0.88	0.0	0.0	14.3	85.7	1.00
3.2 The web software’s contents are relevant for workers to be able to identify aspects related to mental health, as well as look for strategies to strengthen mental health.	0.0	25.0	12.5	62.5	0.75	0.0	0.0	28.6	71.4	1.00
**CVI** ^||^ **- Relevance Dimension**					**0.82**					**1.00**
**4. Environment** 4.1 The environment is suitable for presenting the content.	0.0	12.5	37.5	50.0	0.88	0.0	14.3	28.6	57.1	0.86
4.2 The environment is suitable for understanding workers’ mental health.	0.0	12.5	25.0	62.5	0.88	0.0	14.3	28.6	57.1	0.86
**CVI** ^||^ **- Environment Dimension**					**0.88**					**0.86**

* 1 = Not Relevant

^†^ 2 = Not very relevant

^‡^ 3 = Relevant

^§^ 4 = Highly Relevant

^||^ CVI = Content Validity Index

 The technical validation obtained an overall CVI of 0.97. In relation to the instrument’s dimensions, the one with the lowest CVI was the Functionality Dimension, with a 0.89 CVI ( Table 2 ). 

 It should also be noted that in the Functionality Dimension there was no specific suggestion for *e-LeveMente* , but an improvement in the contextualization before starting to answer the questionnaires was pointed out. No changes were made to this point, as it was considered sufficient contextualization to read and accept the ICF, where the user, before answering the questions, was informed about the research objectives and what their answers consisted of. For technical validation, one cycle of answers was considered sufficient, as no technical changes were pointed out. 

**Table 2 -:** Content validity index and percentage agreement between the judges of the web software technical validation for the mental health promotion in the workplace. Londrina, PR, Brazil, 2023

**Validation items**	**Percentage agreement**				
	1* %	**2** ^†^ **%**	**3** ^‡^ **%**	**4** ^§^ **%**	**CVI** ^||^
**1.** **Ergonomics** 1.1 The user can move from one screen to another quickly.	0.0	0.0	66.7	33.3	1.00
1.2 The data location is kept consistent from one screen to the next.	0.0	0.0	66.7	33.3	1.00
1.3 Text and style features (e.g. underlining, bold, italics) are used appropriately.	0.0	16.7	16.7	66.7	0.83
1.4 Controls and commands are visually differentiated from the information displayed on the screens.	0.0	0.0	33.3	66.7	1.00
1.5 Items selected for triggering are highlighted from the rest.	0.0	0.0	66.7	33.3	1.00
1.6 Error messages are concise and to the point.	0.0	0.0	50.0	50.0	1.00
**CVI** ^||^ **- Ergonomics Dimension**					**0.97**
**2. Features** 2.1 The web software is suitable for its intended purpose.	0.0	0.0	16.7	83.3	1.00
2.2 The web software performs the proposed functions correctly.	0.0	0.0	33.3	66.7	1.00
2.3 The web software makes it possible to generate positive results.	0.0	0.0	16.7	83.3	1.00
**IVC** ^||^ **- Functionality Dimension**					**1.00**
**3. Usability** 3.1 The web software is easy to use.	0.0	16.7	33.3	50.0	0.83
3.2 It’s easy to learn the web software concepts and applications.	0.0	0.0	66.7	33.3	1.00
3.3 The web software makes it easy for workers to apply the content covered.	0.0	16.7	16.7	66.7	0.83
**CVI** ^||^ **- Usability Dimension**					**0.89**
**4. Environment** 4.1 The web software’s response time is adequate for the worker to access the content available on the different screens.	0.0	0.0	33.3	66.7	1.00
4.2 The topic organization on the web software’s different screens is suitable for a good content understanding, as well as easy location of the desired topic.	0.0	0.0	33.3	66.7	1.00
**CVI** ^||^ **- Environment Dimension**					**1.00**

* 1 = Not Relevant

^†^ 2 = Not very relevant

^‡^ 3 = Relevant

^§^ 4 = Highly Relevant

^||^ CVI = Content Validity Index

## Discussion

 The use of technology for health promotion is a growing reality, especially since the COVID-19 pandemic, when digital tools for health management have spread exponentially ^(^ 23 ^)^ . 

 The literature shows that nurse researchers have sought innovative technological solutions for health. Of particular note is the development of software to monitor prenatal care ^(^ 24 ^)^ , injury prevention ^(^ 25 ^)^ , patient education in the preoperative phase of myocardial revascularization ^(^ 26 ^)^ , food consumption assessment ^(^ 27 ^)^ , retirement planning ^(^ 28 ^)^ and to support for family members and close friends of depressed people at potential self-extermination risk ^(^ 29 ^)^ . 

 The web software developed aims to disseminate knowledge and indicate the strategies that support the mental health promotion. In the work environment, it is essential to adopt initiatives to deal with mental health problems. Raising awareness and improving understanding among workers is only part of the effort to reduce stigma, enable them to value their well-being and recognize when to seek help ^(^ 30 ^)^ . 

 A systematic review, which aimed to identify the current evidence for interventions focused on reducing stigma related to mental health problems at work, concluded that a high proportion of the workforce could benefit from interventions designed to reduce stigma related to mental health. Among these, online interventions have shown promise because they are shorter and appear to have the same positive effects as face-to-face interventions ^(^ 31 ^)^ . 

 A systematic review study that sought to characterize the literature and the current state of *mHealth* platforms designed for anxiety or depression available in app stores identified 169 and 179 apps in the Google Play Store and Apple App Store, respectively. The most common platform objective in both searches was treatment, and only 12.3% of the apps mentioned the use of validated tests, guidelines from international organizations, validated therapies or peer-reviewed articles ^(^ 32 ^)^ . 

 The COVID-19 pandemic has highlighted the potential of digital innovations to improve access to and quality of mental health care ^(^ 33 ^)^ . In Brazil, a study that reported on the implementation of a multimodal program that offered preventive actions and mental health treatment to 22,000 workers, demonstrated that the use of the app was a viable strategy on a large scale for mental health screening, offering preventive information with videos for referral to professional care ^(^ 34 ^)^ . 

 Another study evaluating online mindfulness-based interventions to help nursing professionals with mental health self-care found that online education strategies are useful in reducing perceived stress, symptoms of anxiety and depression, as well as boosting participants’ satisfaction with life and work ^(^ 35 ^)^ . 

 Digital innovations in mental health offer great potential, however, some international experts have pointed out that digital interventions are best used as a complement to enrich face-to-face actions, as engagement supported by digital interventions also requires human interaction ^(^ 33 ^)^ . 

 In developing the *e-LeveMente* web software, the resources and content presentation were designed to make it informative, inviting, attractive and easy to use. It should be noted that these aspects have been highlighted as challenges for software ^(^ 28 ^)^ development. Evidence has reported that even interventions that are considered minimalist, such as counseling with online access and support through apps, are associated with lower presenteeism levels ^(^ 36 ^)^ . 

 When designing technological tools, the characteristics, skills and context of users must be assessed ^(^ 37 ^)^ . Stakeholder decision-making is also influenced by the inevitability of going digital. Thus, strengthening the evidence body on digital interventions is important not only to inform whether or not to adopt the decisions, but also how it can become a proficient practice in mental health care ^(^ 10 ^)^ . 

 Digital care represents an advance in the incorporation of technologies into healthcare. This strategy can empower users by self-managing their own health and strengthening preventive and self-care actions ^(^ 38 ^)^ . In addition, the use of information and communication technologies can contribute to innovative and timely interventions that address mental health problems and promote a healthy workforce ^(^ 39 ^)^ . 

 A study evaluating the cost-effectiveness of digital interventions in mental health highlighted that these strategies may be preferred over visiting a healthcare professional. In addition, digital intervention can improve access to services and self-care, enabling continuous care ^(^ 10 ^)^ . 

 A systematic review study evaluating 117 studies and 11,119 randomized participants found that stress among healthcare workers can be tackled at an organizational but also at an individual level. Interventions at the individual level such as focusing on thoughts, feelings, behavior, exercise, relaxation, yoga, acupuncture can reduce stress among healthcare workers up to a year after the intervention ^(^ 40 ^)^ . In this way, it can be inferred that the proposed web software is an accessible tool to be used to promote workers’ mental health in the individual and organizational spheres. 

 The validation stage was essential, as it enabled the web software to be evaluated and improved. Some validation studies have sought to verify the suitability, quality, legitimacy and credibility of an object to be validated based on the opinion of experts on the subject and/or users ^(^ 41 ^)^ . In addition, the development of these technologies has been an opportunity for collaboration between health researchers and technology professionals to design and develop tools to support health professionals and connect them to users ^(^ 9 ^)^ . 

 In this study, it was necessary for the judges to carry out two rounds of revision for content validation, even though the overall CVI and the CVI per dimension were satisfactory. The recommendation was made that if the evaluators identified aspects of the construct that had not been adequately covered, it might need to be revised again ^(^ 17 ^)^ . Another study, which developed and validated an application for prenatal monitoring, also pointed out the need for two-stage validation ^(^ 24 ^)^ . 

 The overall technical and content CVI obtained in this study was considered high, 0.97 and 0.98 respectively. Validation analysis by CVI is widely used. A scoping study that evaluated 881 validation studies in the field of nursing found that, in terms of the analysis type, the use of CVI occurs in approximately 40% of validation studies as one of the most important dimensions in the analysis of a material. This analysis makes it possible to determine validation based on predefined statistical calculations ^(^ 41 ^)^ . 

 Different perceptions were noted in the judges’ evaluations. This is in line with the subjective nature of the method, which takes into account the individuality of the interpretation, which can result in differences in the evaluation ^(^ 41 ^)^ . 

 In the content validation, the judges considered it relevant that the web software enabled self-assessment and immediate classification of mental health in relation to depression, anxiety and stress, and also suggested actions for self-care. Evidence has shown that assessment tools can directly improve users’ mental health by encouraging them to reflect on their own mental health ^(^ 6 ^)^ . 

 Self-assessment is an important step, as early help-seeking for mental health symptoms is still uncommon, yet the literature reports that 70% of people with a clinical or subclinical mental disorder had never sought or received treatment. This data highlighted the indispensability of providing workplace interventions for the prevention, identification and early treatment of depression and anxiety in people who would otherwise not seek help ^(^ 1 ^)^ . 

 Digital technologies have also been recognized as essential for advancing the Sustainable Development Goals ^(^ 7 ^)^ . This study corroborates global initiatives on digital health as a means of promoting access to health, healthy lives and well-being for all, especially through health promotion measures. 

 Although the study’s objectives were achieved, it had limitations related to the *e-LeveMente* validation stages, which were restricted to evaluating the content and technical aspects only with judges, and no validation was carried out with workers. It is therefore suggested that the web software be validated in the workplace at a later date. Another limitation is the scarcity of literature which would allow the results to be compared. 

 It should be noted, however, that the *e-LeveMente* web software contributes to the advancement of scientific knowledge for the mental health promotion in the workplace, as it offers information on mental health in a flexible, less stigmatizing and easily accessible way. It can also help managers and nurses who work in occupational health management, and can be used to assess the workers’ mental health profile, based on access to the database, thus enabling the development of institutional strategies to promote mental health in the workplace. 

## Conclusion

 The web software for promoting mental health in the workplace *e-LeveMente* proved to be valid in terms of its content and technical aspects in the evaluation of judges with expertise in mental health, workers’ health and systems development. It is considered that the agreement between the judges, as well as the suggestions incorporated, demonstrated the potential for use in promoting mental health in the workplace. 

 Thus, although each workplace needs to develop customized solutions, the *e-LeveMente* web software for promoting mental health in the workplace, after validation with workers, could become a tool capable of helping workplaces fulfill their role in promoting mental health. 
