# Supplementary material for: Promoting mental health in the workplace: web software development and validation
Source: Rev Lat Am Enfermagem. 2024 Sep 23;32:e4353. doi: 10.1590/1518-8345.7181.4353 (PMC11421523; doi:10.1590/1518-8345.7181.4353)
Supplement: Translation

# Translation: Promoción de la salud mental en el ambiente laboral: desarrollo y validación de un *software web*

**DOI:** 10.1590/1518-8345.7181.4352

**Published:** 2024-09-23

**Authors:** Evelin Daiane Gabriel Pinhatti, Regina Celia Bueno Rezende Machado, Rosangela Aparecida Pimenta, André Estevam Jaques, Maria do Carmo Fernandez Lourenço Haddad

**Affiliations:** ^1^ Universidade Estadual de Londrina, Londrina, PR, Brasil.; ^2^ Universidade Anhanguera, Unidade Catuaí, Londrina, PR, Brasil.; ^3^ Universidade Estadual de Maringá, Maringá, PR, Brasil.

**Keywords:** Salud Mental, Salud Laboral, Lugar de Trabajo, Tecnología de la Información, Programas Informáticos, Enfermería

## Abstract

**Objetivo:**

desarrollar y validar el contenido y los aspectos técnicos de un *software web* para promover la salud mental en el lugar de trabajo.

**Método:**

estudio metodológico aplicado y de desarrollo tecnológico, realizado en tres etapas: 1) Desarrollo de la *umbrella review* ; 2) Desarrollo del *software web* ; 3) Validación de contenido y técnica realizada por 14 jueces. Los datos fueron sometidos a análisis estadístico descriptivo y cálculo del índice de validez de contenido.

**Resultados:**

a partir de las recomendaciones de las directrices, se definió y extrajo información para desarrollar el *software web* que consta de las siguientes dimensiones: educación en salud mental, apoyo entre compañeros de trabajo, estrategias de promoción y autoevaluación de la salud mental. Para el desarrollo técnico se definieron los objetivos, las funciones generales y la infraestructura tecnológica. Después del desarrollo y las pruebas funcionales, la versión estaba lista para que los jueces realizaran la validación técnica y de contenido. El índice de validez de contenido global fue de 0,98 y para los aspectos técnicos fue de 0,97.

**Conclusión:**

la concordancia entre los jueces sobre el contenido y los aspectos técnicos, al igual que las sugerencias incorporadas, demostraron el potencial del uso del *software web* para promover la salud mental en el lugar de trabajo.

## Puntos destacados

**(1)** La tecnología se utiliza para brindar información sobre salud mental.

**(2)** Permite difundir información de forma flexible y menos estigmatizante.

**(3)** Es útil para prevenir, detectar y enfrentar problemas de salud mental.

**(4)** Ayuda a los directivos y enfermeros ocupacionales a evaluar a los trabajadores.

**(5)** Permite desarrollar estrategias institucionales para promover la salud mental.

## Introducción

 Las enfermedades mentales de los trabajadores se han convertido en un importante problema de salud pública a nivel mundial y son la principal causa de ausentismo laboral por enfermedad e invalidez ^(^ 1 ^)^ . Se estima que el 15% de los adultos experimenta algún tipo de trastorno mental durante su vida laboral. La depresión y la ansiedad se han convertido en las principales causas de incapacidad laboral, ambas han contribuido a que se perdieran anualmente aproximadamente 12 mil millones de días laborales, lo que le ha costado a la economía mundial casi un billón de dólares ^(^ 2 ^)^ . 

 En Brasil, entre 2007 y 2022, se realizaron 17.681 notificaciones de trastornos mentales relacionados con el trabajo ^(^ 3 ^)^ . Debido a esa situación, se han discutido propuestas legislativas para otorgarles incentivos fiscales a las personas jurídicas que implementen programas de salud mental en el ámbito laboral, lo que implica un avance importante para la salud de los trabajadores ^(^ 4 ^)^ . 

 En cuanto a la causalidad, está claro que el trabajo por sí solo no actúa como la principal causa de enfermedad mental. Sin embargo, puede ser un factor causal contribuyente, es decir, actúa como disparador de un trastorno latente o agravante de una enfermedad preexistente ^(^ 5 ^)^ . 

 La Organización Mundial de la Salud (OMS) y la Organización Internacional del Trabajo (OIT) han solicitado que se tomen medidas concretas para abordar las preocupaciones sobre la salud mental de la población trabajadora. Mediante la publicación de directrices, se destaca que el abordaje de la salud mental debe ser amplio e integral, y que es importante que en los ambientes laborales haya un escenario propicio para el cambio, con compromiso para combatir el estigma y la discriminación, coordinando abordajes multisectoriales ^(^ 2 ^)^ . 

 Los problemas de salud mental afectan a las personas en todo el mundo y repercuten en la calidad de vida individual y en la economía de los empleadores ^(^ 6 ^)^ . La evidencia ha demostrado que la implementación de intervenciones en el lugar de trabajo puede promover la salud mental y mejorar el aspecto psicológico del individuo ^(^ 1 ^)^ . Partiendo del supuesto de que algunos problemas de salud mental son evitables, existen argumentos suficientes para que los empleadores inviertan en soluciones preventivas para la salud mental de los trabajadores ^(^ 6 ^)^ . 

 En los últimos años, las tecnologías de salud digital para dispositivos móviles y de escritorio han comenzado a utilizarse para ampliar el acceso a la información. Estas tecnologías pueden aumentar la accesibilidad de las estrategias de salud mental. También pueden reducir los desafíos de la falta de profesionales de la salud al minimizar las barreras que surgen de la necesidad de reuniones presenciales ^(^ 6%5E-%5E@7 ^)^ . Por ende, pueden mejorar las lagunas en la implementación de estrategias y reducir los problemas asociados con la salud mental ^(^ 8 ^)^ . 

 Además, las innovaciones tecnológicas pueden aumentar la concientización y la adherencia a medidas de prevención, educación y apoyo para las personas afectadas por condiciones de salud mental, principalmente en el entorno laboral, donde los enfoques tradicionales tienden a tener baja aceptación ^(^ 6 ^-^ 7 ^,^ 9 ^)^ . El uso de la tecnología también puede considerarse útil y ventajoso para el acceso de los usuarios, dado que algunas personas no buscarían ayuda por miedo a ser estigmatizadas ^(^ 9 ^)^ . 

 El uso de tecnologías para prevenir o mejorar problemas de salud mental puede incluir acciones que utilizan *software* , a los que se puede acceder a través de computadoras, *tablets, smartphones* , equipos audiovisuales, robots y otros dispositivos. Los dispositivos cuentan con recursos para recolectar, almacenar y recuperar información, además de guiar a los usuarios en la realización de actividades terapéuticas ^(^ 10 ^)^ . 

 En Brasil, la literatura muestra que la producción de *software* está orientada a dirigir la organización de los servicios de salud ^(^ 11%5E-%5E@12 ^)^ , la gestión de la atención de enfermería ^(^ 13 ^)^ , la enseñanza de enfermería ^(^ 14%5E-%5E@15 ^)^ y el autocuidado de los pacientes ^(^ 16 ^)^ . 

 Considerando lo expuesto anteriormente y el vacío que hay en la producción de conocimiento científico nacional y en el desarrollo de tecnologías para promover la salud mental de los trabajadores, la pregunta de investigación del presente estudio es: ¿el *software web* desarrollado para promover la salud mental en el ambiente laboral tiene validez de contenido y técnica? 

 Para responder a esta pregunta de investigación, el objetivo de este estudio fue desarrollar y validar el contenido y los aspectos técnicos de un *software web* para promover la salud mental en el ambiente laboral. 

## Método

### Diseño del estudio

 Se trata de un estudio metodológico aplicado y de desarrollo tecnológico, destinado a desarrollar o mejorar métodos para la obtención, organización o análisis de datos, enfocado en el desarrollo, la validación y la evaluación de herramientas ^(^ 17 ^)^ . 

### Periodo

El estudio fue desarrollado entre enero de 2021 y diciembre de 2022.

### Procedimientos del estudio

 El estudio se realizó en tres etapas: 1) Para fundamentar la elaboración del *software web* se realizó un estudio *umbrella review* con el fin de identificar las recomendaciones de las directrices para promover la salud mental en el ambiente laboral; 2) Desarrollo del *software web* ; 3) Validación de contenido y técnica del *software web* por parte de los jueces. 

En la primera etapa, se realizó una búsqueda de revisiones sistemáticas que identificarán las recomendaciones de directrices para promover la salud mental en el ambiente laboral.

 La búsqueda se llevó a cabo en cinco bases de datos: *American Psychological Association* (PsycINFO), *Cochrane Library* , EMBASE, *National Library of Medicine/National Institutes of Health* (MEDLINE a través de PubMed) y *Scopus* . Se utilizaron los descriptores “ *mental health”, “worpkplace”, “guidelines”, “systematic review”* . El protocolo de la *umbrella review* está registrado en la plataforma Prospero CRD42023461845. 

Los resultados se agruparon en tres categorías de recomendaciones, a saber: a) Recomendaciones de prevención primaria – protección del trabajador; b) Recomendaciones de prevención secundaria – promover la salud mental del trabajador; y c) Recomendaciones de prevención terciaria: apoyar, monitorear y readaptar al trabajador cuando vuelve al trabajo después de haber estado ausente.

 A partir de la evaluación de las recomendaciones descritas en las directrices, se definió y extrajo información para que se insertara en el *software web* el siguiente contenido: concientización sobre la salud mental, apoyo social a los compañeros de trabajo, estrategias de promoción y autoevaluación de la salud mental. La autoevaluación se basó en la aplicación de la Escala de Depresión, Ansiedad y Estrés (DASS-21), ya traducida y válida para Brasil ^(^ 18 ^)^ . 

 En la segunda etapa, es decir, para crear el *software web* , se utilizaron los pasos metodológicos de la ingeniería de *software* ^(^ 19 ^)^ . Este modelo permite el desarrollo de un *software web* a partir de una serie de versiones evolucionadas que incluyen las fases de comunicación, planificación, modelado, construcción, evaluación, pruebas de funcionamiento y versión para validación. Además, permite realizar las adaptaciones que sean necesarias a lo largo de la vida del *software* ^(^ 19 ^)^ . 

 Para el desarrollo del *software web* , se contactó a un profesional con experiencia en el desarrollo de sistemas, por medio de reuniones sistemáticas, en las que se definió la planificación, que incluyó los principios requisitos y los recursos del *software web* , además de un cronograma preliminar para el desarrollo. 

 El modelado del *software web* se estableció mediante un diagrama de flujo con la interfaz de las pantallas. Para la construcción se delineó el *layout* visible para el usuario, que definió la disposición de las pantallas y los íconos de acceso. El *software web* fue desarrollado basándose en los conceptos de *Responsive Web Design* , que permite que se acceda al mismo desde una computadora de escritorio, *tablet* o *smartphone* cambiando el *layout* sobre el tamaño de la pantalla, que facilita su visualización. La arquitectura utilizada fue *Content Management System* (CMS). Esta herramienta cuenta con varias funciones con el objetivo de facilitar la creación y edición del contenido. El lenguaje de programación utilizado fue *Personal Home Page* (PHP), *Hyper Text Markup Language* (HTML5), *Cascading Style Sheets* (CSS3) y *Javascript* ^(^ 20 ^)^ . 

 Para crear el *frontend* del *software web* , que incluye la interfaz visual para el usuario, se utilizó el programa *Adobe Photoshop* ^®^ , *Javascript* con *framework* Vue.js, HTML5 y CSS3. Para el *backend* , que incluye el procesamiento y almacenamiento de los datos, se utilizó el administrador de base de datos MySQL. Se usó un *framework* para administrar el contenido, el cual se administra a través del acceso al panel administrativo de la herramienta, lo que les facilita a los investigadores actualizar el contenido. El *framework* se alojó en el servidor Linux/Apache. Durante la fase de prueba, se verificó si era necesario realizar ajustes en la interfaz y la funcionalidad testeando los *links* y formularios ^(^ 20 ^)^ . 

 La tercera etapa consistió en la validación del contenido y de los aspectos técnicos por parte de los jueces, siguiendo la recomendación de incluir seis o más expertos ^(^ 21 ^)^ . La selección de los jueces se realizó a través de la Plataforma Lattes, mediante muestreo no probabilístico intencional, y según los criterios de inclusión. 

Para el juez de contenido se establecieron los siguientes criterios de inclusión: contar como mínimo con título de doctorado; ser autor/orientador de estudios relacionados con la salud ocupacional y/o salud mental; dicta/dictó clases/conferencias enfocadas en la salud ocupacional y/o la salud mental; participa/participó en grupos/proyectos relacionados con la salud ocupacional y/o la salud mental.

 Para el juez técnico se establecieron los siguientes criterios: tener título de especialista; autor/orientador de estudios relacionados con el desarrollo de *software* ; dicta/dictó clases/conferencias enfocadas en el desarrollo de *softwar* e; participa/participó en grupos/proyectos relacionados con el desarrollo del *software* . 

 Fueron invitados 22 jueces, mediante carta de invitación por correo electrónico. De los 22, ocho no respondieron al contacto, lo que dio como resultado una muestra de 14 jueces, ocho de los cuales eran enfermeros y seis especialistas en desarrollo de *software* . 

### Instrumentos utilizados para recopilar información

 Para la recolección de datos se crearon dos formularios diferentes utilizando la herramienta *Google Forms* . En el formulario se incluyeron el Formulario de Consentimiento Libre e Informado (FCLI) y el *link* para acceder al *software web* , junto con los ítems del instrumento de validación. Se fijó un plazo de 15 días para la evaluación y cumplimentación del instrumento, y se realizaron dos intentos más para reenviarlo con el mismo plazo de respuesta. La no cumplimentación de los formularios se consideró intención de no participar. 

 El instrumento de validación de contenido estaba compuesto por 13 afirmaciones que incluían ítems sobre objetivos, contenido, relevancia y ambiente. El instrumento de validación técnica estaba compuesto por 14 enunciados que incluían ítems sobre ergonomía, funcionalidad, usabilidad y ambiente. Cada una de las dimensiones contenía ítems que se evaluaban mediante una escala Likert de cuatro puntos, distribuidos en no relevante, poco relevante, relevante y muy relevante. Al final de cada dimensión se dispuso un espacio para que los jueces pudieran hacer sugerencias. Los instrumentos fueron adaptados de un estudio de validación de *software web* denominado *Aposentar-se com Saúde* (Jubilarse con Salud), que fue adaptado para el tema promoción de la salud mental en el ambiente laboral ^(^ 22 ^)^ . 

### Análisis de los datos

 El análisis de los datos obtenidos se realizó mediante estadística descriptiva, utilizando valores de frecuencia absoluta y relativa, con ayuda de los programas *Microsoft Office Excel* ^®^ y *Statistical Package for the Social Sciences* (SPSS) ^®^ versión 25.0. Para llevar a cabo el análisis de validez técnica y de contenido se aplicó el cálculo del Índice de Validez de Contenido (IVC), considerando la proporción de respuestas relevantes o muy relevantes, dividida por el total de respuestas de cada ítem. El IVC de las dimensiones del instrumento se obtuvo de la suma de los IVC de las dimensiones, dividido por el número de ítems. Finalmente, para obtener el IVC global se realizó la suma de los IVC individuales divididos por el número total de ítems del instrumento. El valor mínimo que se adoptó para el IVC fue 0,78 ^(^ 21 ^)^ . Las sugerencias enviadas por los jueces fueron organizadas y analizadas según las dimensiones del instrumento. 

### Aspectos éticos

Este estudio cumple con la Resolución N° 466, del 12 de diciembre de 2012, y fue previamente aprobado por el Comité de Ética en Investigación (CEI) de la institución, según el Dictamen nº 3.588.273.

## Resultados

 El *software web* se denominó *e-LeveMente* , está alojado en un servidor *web* y se puede acceder a él a través del *link* https://www.elevemente.com . Para el desarrollo se realizó una reunión con los desarrolladores, con el fin de definir los objetivos y las funciones generales, a partir de la cual se creó la infraestructura tecnológica con un diagrama de flujo del funcionamiento del *software* , en el que se definieron la secuencia del contenido, la selección de los medios de comunicación, el modo de integración de las pantallas y sus funciones, como se puede ver en la Figura 1 . 

**Figura 1 -:** Diagrama de flujo del funcionamiento del software web para promover la salud mental en el ambiente laboral. Londrina, PR, Brasil, 2023

 Para el desarrollo técnico se definieron el funcionamiento de la pantalla de inicio y el *layout* de presentación ( Figura 2 ). El contenido se presentó en formato de texto, vídeos e imágenes, con el objetivo de brindarle al usuario un entorno atractivo. Al acceder a *e-LeveMente* , en la esquina superior derecha de la pantalla se encuentran las secciones principales y los recursos, que incluyen: página de inicio, sección sobre *cómo apoyar a alguien* , sesión para *promover tu salud mental* y el ícono *evalúa tu salud mental* ( Figura 2 ). 

La página de inicio incluye información sobre la definición de salud mental, los problemas comunes de salud mental, factores que influyen en la salud mental, signos y síntomas de problemas de salud mental y las consecuencias de los problemas de salud mental en el trabajo.

 La sesión sobre *cómo apoyar a alguien* , proporciona información sobre cómo propiciar una conversación inicial y abordar el tema, qué preguntar, la elección del lugar adecuado, la importancia de mantener la confidencialidad y tranquilizar a la persona, cómo animar a la persona a hablar y buscar ayuda profesional, considerando las estrategias para establecer una comunicación efectiva. 

**Figura 2 -:** Representación gráfica de la pantalla de inicio y del contenido del *software web* para promover la salud mental en el ambiente laboral. Londrina, PR, Brasil, 2023

 En la sección *promover tu salud mental* , hay contenido disponible para el autocuidado. Las estrategias incluyen orientaciones como: la importancia de mantenerse activo, establecer un equilibrio entre el trabajo y la vida privada, cultivar relaciones saludables, advertencia sobre los riesgos de fumar, beber y consumir drogas para reducir el malestar relacionado con la salud mental, la importancia de comer bien, técnicas de relajación, hábitos para mejorar la calidad del sueño, habilidades positivas de afrontamiento, prácticas saludables en el lugar de trabajo, advertencia de no automedicarse en caso de signos y síntomas relacionados con la salud mental, prácticas para una comunicación asertiva, sugerencias de aplicaciones que ayudan a gestionar la salud mental e información sobre dónde buscar ayuda profesional. 

 En todas las secciones, el ícono *evalúa tu salud mental* está disponible en la esquina superior derecha de la pantalla. Al seleccionar *iniciar evaluación* , el usuario es dirigido a una pantalla donde visualiza el FCLI y, luego de leerlo y aceptarlo, es dirigido a la pantalla que tiene el cuestionario de caracterización sociodemográfica y ocupacional. Las preguntas en la *interfaz* del *software web* son obligatorias y le permiten pasar a la siguiente pantalla, después de haberlas completado todas. 

En la siguiente pantalla se encuentra disponible el instrumento de autoevaluación y seguimiento de las condiciones de salud mental. Antes de que el usuario empiece a responder se emite la siguiente información: “El cuestionario es una medición objetiva, confiable y clara para que el usuario comprenda cómo se siente. El resultado de la evaluación no indica un diagnóstico concluyente, sino el punto de partida para incentivar el autocuidado o la búsqueda de ayuda profesional”.

 Las preguntas están disponibles en tres pantallas con siete preguntas, de modo que el usuario puede ingresar una opción de respuesta para cada pregunta, y esto le permite pasar de una pantalla a otra directamente, utilizando los botones para avanzar o volver. Una vez que el usuario responde todas preguntas del cuestionario, el *software* calcula las puntuaciones según las recomendaciones de la escala, le asigna un puntaje a cada individuo y genera una página con los resultados. El resultado se entrega dividido en tres factores: depresión, ansiedad y estrés, y se clasifica como normal, mínimo, moderado, severo y muy severo. 

 En función de la puntuación obtenida se dan estrategias para promover la salud mental e información para buscar ayuda profesional, a las que se accede a través del *link* disponible ( Figura 3 ). Las respuestas a los cuestionarios se almacenan en la base de datos de *e-LeveMente* y se puede acceder a ellas a través del área administrativa y exportarlas para futuros análisis. 

**Figura 3 -:** Representación gráfica del cuestionario de autoevaluación de salud mental y la pantalla de resultados del *software web* para promover la salud mental en el ambiente laboral *.* Londrina, PR, Brasil, 2023

 Cabe destacar que, durante el desarrollo de *e-LeveMente* , se realizaron pruebas para identificar posibles errores en la estética, el contenido y los *links* , con el objetivo de crear una herramienta que cuente con la funcionalidad adecuada. Después de realizar las pruebas funcionales, la versión estaba disponible para que los jueces lleven a cabo la validación de contenido y técnica. 

Para la validación del contenido, se obtuvo retroalimentación de ocho jueces de contenido. El tiempo medio de graduados era de 25,4 años. De ellos, el 25% tenía posdoctorado, el 87,5% tenía experiencia en salud ocupacional y el 87,5% tenía experiencia en salud mental.

 En el primer ciclo de validación de contenido, el IVC global fue de 0,85. Con respecto a las dimensiones en las que se distribuyeron los ítems, el que presentó menor IVC fue el dominio contenido, con un IVC de 0,79. A pesar de presentar un IVC aceptable, cinco de los ítems evaluados (2.2; 2.3; 2.5; 2.6 y 3.2) no alcanzaron el IVC mínimo adoptado en este estudio. Por consiguiente, dado que los jueces hicieron sugerencias para mejorar la calidad del contenido de *e-LeveMente* , se las evaluó para realizar las reformulaciones. 

 En relación a las consideraciones de los jueces, en la Dimensión Objetivo, sugirieron que la información sobre la promoción de la salud mental fuera pública, dado que de lo contrario el usuario sólo tendría acceso a ella después de completar el cuestionario de autoevaluación. Esta sugerencia fue aceptada y se creó la sección *promover tu salud mental* en el menú principal. 

 En la Dimensión Contenido los jueces sugirieron realizar cambios en el estilo de la fuente y revisión gramatical de la información. En el cuestionario de caracterización, en el ítem sexo, sugirieron incluir la opción *prefiero no contestar* . Con respecto al cuestionario de autoevaluación de salud mental, recomendaron incluir la referencia completa, y no solo el nombre de los autores. 

 En las estrategias de promoción de la salud mental recomendaron sustituir el término “fomentar relaciones” por “cultivar relaciones sanas” e incluir la palabra “evite” antes de: fumar, beber y consumir drogas. Además, sugirieron incluir información sobre automedicación, sueño y comunicación asertiva. En técnicas de relajación, la sugerencia fue incluir algunas aplicaciones gratuitas de relajación y meditación para *Android* y *iPhone Operating System* (iOS). 

 En la Dimensión Relevancia, se destacó que el formato utilizado para presentar el contenido sobre las consecuencias de los problemas de salud mental en el trabajo era excelente, al igual que las estrategias para ayudar a los trabajadores a hablar sobre su salud mental y las directrices pertinentes en la *checklist* de comunicación. También se consideró relevante, en la evaluación de su salud mental, brindar orientación sobre los resultados y las estrategias para promover la salud mental. Algunos jueces insistieron en que se estaba logrando identificar la salud mental, pero que era necesario mejorar las estrategias, como se indicó en la Dimensión Contenido. 

En la Dimensión Ambiente, los jueces destacaron que los colores suaves y las figuras eran apropiadas y tenían un excelente diseño. El material es de fácil acceso, tiene contenido significativo y relevante para la salud mental del trabajador, videos bien diseñados, claros y con la capacidad de transmitir la información y orientación necesarias. Sólo recomendaron diversificar las melodías presentes en los videos y reducir la velocidad de reproducción, para que el usuario pueda asimilar la información con mayor facilidad.

 Cabe destacar que las sugerencias que realizaron los jueces fueron evaluadas e incorporadas a la nueva versión de *e-LeveMente* para su validación. Por ello, la versión adecuada fue enviada a un nuevo ciclo de validación. En este caso, se obtuvo retroalimentación de siete jueces y el IVC general fue de 0,98, como se muestra en la Tabla 1 . 

 En la validación técnica se obtuvo retroalimentación de seis jueces. El tiempo medio de graduados era de 11,8 años, tenían títulos en Análisis de Sistemas, Informática, Ingeniería Informática, Ingeniería de Desarrollo de *Software* y Redes de Computadoras. De ellos, el 16,7% tenía título de doctorado, el 33,3% tenía título de maestría y el 50% era especialista. 

**Tabla 1 -:** Índice de Validez de Contenido y porcentaje de concordancia de los jueces que validaron el contenido del *software web* para promover la salud mental en el ambiente laboral. Londrina, PR, Brasil, 2023

Ítems de validación	**1° Porcentaje de concordancia**					**2° Porcentaje de concordancia**				
	1* %	**2** ^†^ **%**	**3** ^‡^ **%**	**4** ^§^ **%**	**IVC** ^||^	1* %	**2** ^†^ **%**	**3** ^‡^ **%**	**4** ^§^ **%**	**IVC** ^||^
**1. Objetivos** 1.1 Los objetivos del *software web* son consistentes con la promoción de la salud mental.	12,5	0,0	37,5	50,0	0,88	0,0	0,0	28,6	71,4	1,00
1.2 El *software web* facilita la comprensión del tema promoción de la salud mental.	0,0	0,0	37,5	62,5	1,00	0,0	0,0	28,6	71,4	1,00
1.3 Los objetivos propuestos son adecuados para la implementación.	0,0	12,5	25,0	62,5	0,88	0,0	0,0	28,6	71,4	1,00
**IVC** ^||^ **- Dimensión Objetivos**					**0,92**					**1,00**
**2. Contenido** 2.1 El contenido del *software web* coincide con los objetivos.	0,0	12,5	37,5	50,0	0,88	0,0	0,0	28,6	71,4	1,00
2.2 El contenido del *software web* es suficiente para alcanzar los objetivos propuestos.	0,0	25,0	62,5	12,5	0,75	0,0	0,0	57,1	42,9	1,00
2.3 El contenido del *software web* logra con precisión el abordaje del tema.	0,0	37,5	50,0	12,5	0,63	0,0	0,0	42,9	57,1	1,00
2.4 La información presentada es correcta.	0,0	0,0	62,5	37,5	1,00	0,0	0,0	14,3	85,7	1,00
2.5 La forma de redacción es adecuada para los iferentes niveles de conocimiento de los trabajadores de las instituciones de salud.	0,0	25,0	25,0	50,0	0,75	0,0	0,0	28,6	71,4	1,00
2.6 El contenido facilita la comprensión de las diferentes dimensiones de la promoción de la salud mental.	0,0	25,0	37,5	37,5	0,75	0,0	0,0	42,9	57,1	1,00
**IVC** ^||^ **- Dimensión Contenido**					**0,79**					**1,00**
**3. Relevancia** 3.1 El contenido aborda aspectos clave que hay que explorar en la promoción de la salud mental.	0,0	12,5	25,0	62,5	0,88	0,0	0,0	14,3	85,7	1,00
3.2 El contenido del *software web* es relevante para que los trabajadores puedan identificar aspectos relacionados con la salud mental y para buscar estrategias que fortalezcan la salud mental.	0,0	25,0	12,5	62,5	0,75	0,0	0,0	28,6	71,4	1,00
**IVC** ^||^ **- Dimensión Relevancia**					**0,82**					**1,00**
**4. Ambiente** 4.1 El ambiente es adecuado para presentar el contenido.	0,0	12,5	37,5	50,0	0,88	0,0	14,3	28,6	57,1	0,86
4.2 El ambiente es adecuado para comprender la salud mental del trabajador.	0,0	12,5	25,0	62,5	0,88	0,0	14,3	28,6	57,1	0,86
**IVC** ^||^ **- Dimensión Ambiente**					**0,88**					**0,86**

* 1 = No Relevante

^†^ 2 = Poco Relevante

^‡^ 3 = Relevante

^§^ 4 = Muy Relevante

^||^ IVC = Índice de Validez de Contenido

 La validación técnica obtuvo un IVC global de 0,97. En cuanto a las dimensiones del instrumento, la que presentó menor IVC fue la Dimensión Funcionalidad, cuyo IVC fue de 0,89 ( Tabla 2 ). 

 Además, cabe destacar que en la Dimensión Funcionalidad los jueces no hicieron una sugerencia específica sobre el *e-LeveMente* , pero señalaron que habría que mejorar la contextualización antes de comenzar a responder los cuestionarios. No se realizaron cambios en ese aspecto, porque se consideró que la lectura y aceptación del FCLI era contextualización suficiente, dado que al usuario antes de responder las preguntas se le informaban los objetivos de la investigación y en qué consistían las respuestas. Para la validación técnica, se consideró suficiente un ciclo de respuestas, dado que no sugirieron cambios técnicos. 

**Tabla 2 -:** Índice de Validez de Contenido y porcentaje de concordancia de los jueces técnicos que validaron el *software web* para promover la salud mental en el trabajo. Londrina, PR, Brasil, 2023

Ítems de validación	**Porcentaje de concordancia**				
	1* %	**2** ^†^ **%**	**3** ^‡^ **%**	**4** ^§^ **%**	**IVC** ^||^
**1.Ergonom** í **a** 1.1 El usuario puede pasar de una pantalla a otra rápidamente.	0,0	0,0	66,7	33,3	1,00
1.2 La ubicación de los datos se mantiene de forma consistente de una pantalla a la otra.	0,0	0,0	66,7	33,3	1,00
1.3 Los textos y los recursos de estilo (p. ej., subrayado, negrita, cursiva) se utilizan adecuadamente.	0,0	16,7	16,7	66,7	0,83
1.4 Los controles y comandos se diferencian visualmente de la información presentada en las pantallas.	0,0	0,0	33,3	66,7	1,00
1.5 Los ítems seleccionados para accionar se destacan de los demás.	0,0	0,0	66,7	33,3	1,00
1.6 Los mensajes de error son concisos y objetivos.	0,0	0,0	50,0	50,0	1,00
**IVC** ^||^ **- Dimensión Ergonomía**					**0,97**
**2. Funcionalidad** 2.1 El *software web* es adecuado para los fines para los que está destinado.	0,0	0,0	16,7	83,3	1,00
2.2 El *software web* realiza correctamente las funciones propuestas.	0,0	0,0	33,3	66,7	1,00
2.3 El *software web* permite generar resultados positivos.	0,0	0,0	16,7	83,3	1,00
**IVC** ^||^ **- Dimensión Funcionalidad**					**1,00**
**3. Usabilidad** 3.1 El *software web* es fácil de usar.	0,0	16,7	33,3	50,0	0,83
3.2 Es fácil aprender los conceptos y aplicaciones del *software web* .	0,0	0,0	66,7	33,3	1,00
3.3 El *software web* les permite a los trabajadores aplicar fácilmente el contenido abordado.	0,0	16,7	16,7	66,7	0,83
**IVC** ^||^ **- Dimensión Usabilidad**					**0,89**
**4. Ambiente** 4.1 El tiempo de respuesta del *software web* es adecuado para que el trabajador acceda al contenido disponible en las diferentes pantallas.	0,0	0,0	33,3	66,7	1,00
4.2 La organización de los temas en las diferentes pantallas del *software web* es adecuada para lograr una buena comprensión del contenido y una fácil localización del tema deseado.	0,0	0,0	33,3	66,7	1,00
**IVC** ^||^ **- Dimensión Ambiente**					**1,00**

* 1 = No Relevante

^†^ 2 = Poco Relevante

^‡^ 3 = Relevante

^§^ 4 = Muy Relevante

^||^ IVC = Índice de Validez de Contenido

## Discusión

 El uso de la tecnología para promover la salud viene en aumento, especialmente después de la pandemia de COVID-19, momento en que las herramientas digitales para la gestión de la salud se extendieron exponencialmente ^(^ 23 ^)^ . 

 En la literatura se observa que los enfermeros investigadores han buscado soluciones tecnológicas innovadoras para la salud. Entre las que se destaca el desarrollo de *software* para el seguimiento prenatal ^(^ 24 ^)^ , la prevención de lesiones ^(^ 25 ^)^ , la educación del paciente en el período preoperatorio de revascularización miocárdica ^(^ 26 ^)^ , la evaluación del consumo de alimentos ^(^ 27 ^)^ , la planificación de la jubilación ^(^ 28 ^)^ y para brindarles apoyo a los familiares y amigos cercanos de personas deprimidas en riesgo potencial de suicidio ^(^ 29 ^)^ . 

 El objetivo del *software web* desarrollado es difundir conocimiento e indicar estrategias que contribuyan a promover la salud mental. En el mundo del trabajo es fundamental adoptar iniciativas para abordar los problemas de salud mental. Crear conciencia y mejorar la comprensión de los trabajadores es solo parte de los esfuerzos para reducir el estigma, capacitarlos para que valoren su bienestar y reconozcan cuándo buscar ayuda ^(^ 30 ^)^ . 

 Una revisión sistemática, cuyo objetivo era identificar evidencia actual de intervenciones centradas en reducir el estigma relacionado con los problemas de salud mental en el trabajo, concluyó que las intervenciones destinadas a reducir el estigma relacionado con la salud mental serían beneficiosas para una alta proporción de la fuerza laboral. Las intervenciones *online* demostraron ser prometedoras porque son más cortas y parece que tienen los mismos efectos positivos que las intervenciones presenciales ^(^ 31 ^)^ . 

 Un estudio de revisión sistemática, que buscó caracterizar la literatura y el estado actual de las plataformas *mHealth* diseñadas para la ansiedad o la depresión disponibles en las tiendas de aplicaciones, identificó 169 y 179 aplicaciones en *Google Play Store* y *Apple App Store* , respectivamente. El objetivo más común de las plataformas, en ambas búsquedas, fue el tratamiento y sólo en el 12,3% de las aplicaciones se mencionó el uso de pruebas validadas, directrices de organismos internacionales, terapias validadas o artículos revisados por pares ^(^ 32 ^)^ . 

 La llegada del COVID-19 aumentó el potencial de las innovaciones digitales para mejorar el acceso y la calidad de la atención en salud mental ^(^ 33 ^)^ . En Brasil, un estudio que documentó la implementación de un programa multimodal que les brindó acciones preventivas y tratamiento de salud mental a 22 mil trabajadores, demostró que el uso de una aplicación era una estrategia viable a gran escala para la detección de la salud mental, brindó información preventiva con videos para orientarlos sobre la atención profesional ^(^ 34 ^)^ . 

 Otro estudio que evaluó intervenciones *online* basadas en *mindfulness* para ayudar a los profesionales de enfermería en el autocuidado de la salud mental, demostró que las estrategias de educación *online* son útiles para reducir el estrés percibido, los síntomas de ansiedad y depresión, y, además, promueve la satisfacción con la vida y el trabajo de los participantes ^(^ 35 ^)^ . 

 Las innovaciones digitales en salud mental tienen potencial; sin embargo, algunos expertos internacionales destacaron que las intervenciones digitales se utilizan mejor como complemento para enriquecer las acciones presenciales, dado que la participación en intervenciones digitales también requiere interacción humana ^(^ 33 ^)^ . 

 En el desarrollo del *software web* *e-LeveMente* , los recursos y la presentación del contenido se diseñaron para que sea informativo, atractivo y fácil de usar. Cabe destacar que estos aspectos han sido todo un desafío para el desarrollo de *software* ^(^ 28 ^)^ . La evidencia ha informado que incluso las intervenciones que se consideran minimalistas, como el asesoramiento con acceso y apoyo *online* a través de aplicaciones, se asocian con niveles más bajos de presentismo ^(^ 36 ^)^ . 

 Cuando se diseñan herramientas tecnológicas hay que evaluar las características, las habilidades y el contexto de los usuarios ^(^ 37 ^)^ . La toma de decisiones de las partes interesadas también se ve influenciada por el hecho de que la digitalización es inevitabilidad. Por lo tanto, es importante robustecer la evidencia sobre las intervenciones digitales, no sólo para informar si se deben adoptar o no las decisiones, sino también cómo se puede transformar en una práctica competente para la atención en salud mental ^(^ 10 ^)^ . 

 La atención digital representa un avance en la incorporación de tecnologías a la atención para la salud. Esta estrategia puede propiciar el empoderamiento de los usuarios a través de la autogestión de su propia salud y el fortalecimiento de acciones preventivas y de autocuidado ^(^ 38 ^)^ . Además, el uso de tecnologías de la información y la comunicación puede aportar intervenciones innovadoras y oportunas que aborden los problemas de salud mental y promuevan una fuerza laboral saludable ^(^ 39 ^)^ . 

 Un estudio que evaluó el costo-beneficio de las intervenciones digitales de salud mental destacó que, probablemente, estas estrategias son preferibles, en vez de visitar a un profesional de la salud. Además, la intervención digital puede mejorar el acceso a los servicios y el autocuidado, lo que permite una atención continua ^(^ 10 ^)^ . 

 En un estudio de revisión sistemática que evaluó 117 estudios y 11.119 participantes aleatorizados, se identificó que el estrés en los profesionales de la salud se puede combatir a nivel organizacional, pero también a nivel individual. Las intervenciones a nivel individual, como concentrarse en los pensamientos, los sentimientos y el comportamiento, como el ejercicio, la relajación, el yoga y la acupuntura, pueden reducir el estrés de los profesionales de la salud hasta un año después de la intervención ^(^ 40 ^)^ . Por lo tanto, se puede inferir que el *software web* propuesto es una herramienta accesible que se puede utilizar para promover la salud mental de los trabajadores a nivel individual y organizacional. 

 En cuanto a la etapa de validación, fue una fase fundamental, dado que permitió evaluar y mejorar el *software web* . Algunos estudios de validación buscaron verificar la idoneidad, calidad, legitimidad y credibilidad de un objeto a ser validado a partir de la opinión de expertos en la materia y/o usuarios ^(^ 41 ^)^ . Además, el desarrollo de estas tecnologías ha brindado una oportunidad de colaboración entre investigadores de la salud y profesionales de la tecnología para diseñar y desarrollar herramientas que ayuden a los profesionales de la salud y los conecten con los usuarios ^(^ 9 ^)^ . 

 En este estudio fue necesario que los jueces realizaran dos ciclos de revisión para validar el contenido, a pesar de que se obtuvo un IVC global y por dimensiones satisfactorio. Se siguió la recomendación de que, si los evaluadores identificaban aspectos del constructo que no se habían contemplado adecuadamente, podría ser necesaria una nueva revisión ^(^ 17 ^)^ . Otro estudio, que desarrolló y validó una aplicación para el seguimiento prenatal, también destacó que era necesario llevar a cabo la validación en dos etapas ^(^ 24 ^)^ . 

 El IVC global técnico y de contenido obtenido en este estudio se consideró alto, 0,97 y 0,98 respectivamente. El análisis de validación mediante IVC es ampliamente utilizado, en un estudio de revisión de alcance, que evaluó 881 estudios de validación en el área de Enfermería, comprobó que, en lo que respecta al tipo de análisis, en aproximadamente el 40% de los estudios de validación se usa el IVC como una de las dimensiones más importantes en el análisis de un material. Este análisis permite determinar la validación en base a cálculos estadísticos predefinidos ^(^ 41 ^)^ . 

 Se observaron diferentes percepciones en las evaluaciones realizadas por los jueces. Ello coincide con la subjetividad característica del método, que considera la interpretación individualidad, lo que puede dar como resultado diferencias en la evaluación ^(^ 41 ^)^ . 

 En la validación de contenido, los jueces consideraron que era relevante que el *software web* permitiera la autoevaluación y clasificación inmediata de la salud mental con respecto a la depresión, la ansiedad y el estrés, y que sugiriera acciones para el autocuidado. La evidencia ha demostrado que las herramientas de evaluación pueden mejorar directamente la salud mental de los usuarios, al animarlos a reflexionar sobre su propia salud mental ^(^ 6 ^)^ . 

 La autoevaluación es una etapa, un paso importante, ya que la búsqueda temprana de ayuda para tratar los síntomas de salud mental aún es poco común; sin embargo, en la literatura se informa que el 70% de las personas que tenían un trastorno mental clínico o subclínico nunca habían buscado ni recibido tratamiento. Estos datos denotan que es imprescindible que se implementen intervenciones en el lugar de trabajo para prevenir, identificar y tratar de forma temprana la depresión y la ansiedad de las personas que de otro modo no buscarían ayuda ^(^ 1 ^)^ . 

 También se ha reconocido que las tecnologías digitales son esenciales para promover los objetivos de desarrollo sostenible ^(^ 7 ^)^ . Este estudio coincide con las iniciativas globales sobre salud digital, como medio para promover el acceso a la salud, la vida sana y el bienestar para todos, principalmente con medidas para promover la salud. 

 Si bien se alcanzaron los objetivos del estudio, el mismo tiene limitaciones relacionadas con las etapas de validación del *e-LeveMente* , que se restringieron a realizar la evaluación de contenido y de los aspectos técnicos sólo con jueces, es decir, no se realizó una validación con trabajadores. Por lo que se sugiere que el *software web* sea posteriormente validado en el ambiente laboral. Otra limitación es la escasez de literatura que permitiera comparar los resultados. 

 Sin embargo, cabe destacar que el *software web* *e-LeveMente* contribuye al avance del conocimiento científico para la promoción de la salud mental en el ambiente laboral, ya que brinda información sobre salud mental de manera flexible, menos estigmatizante y de fácil acceso. También puede ayudar a los directivos y enfermeros que trabajan en la gestión de la salud del trabajador, dado que puede ser utilizado para evaluar el perfil de la salud mental de los trabajadores, mediante el acceso a la base de datos y, de esa forma, permite el desarrollo de estrategias institucionales para promover la salud mental en el ambiente laboral. 

## Conclusión

 La evaluación de los jueces con experiencia en salud mental, salud del trabajador y desarrollo de sistemas validaron el contenido y los aspectos técnicos del *software web* para promover la salud mental en el ambiente laboral *e-LeveMente* . Se considera que la concordancia de los jueces, así como las sugerencias incorporadas, demostraron el potencial de uso que tiene para promover la salud mental en el ambiente laboral. 

 Por lo tanto, cabe destacar que, si bien cada lugar de trabajo debe crear soluciones personalizadas, el *software web* para la promoción de la salud mental en el lugar de trabajo *e-LeveMente* , después de ser validado por los trabajadores, podría convertirse en una herramienta que contribuya a que los lugares de trabajo cumplan con su parte en la promoción de la salud mental. 

# Translation: **Promoção da saúde mental no ambiente laboral: desenvolvimento e validação de um *web software*** *

**DOI:** 10.1590/1518-8345.7181.4354

**Published:** 2024-09-23

**Authors:** Evelin Daiane Gabriel Pinhatti, Regina Celia Bueno Rezende Machado, Rosangela Aparecida Pimenta, André Estevam Jaques, Maria do Carmo Fernandez Lourenço Haddad

**Affiliations:** ^1^ Universidade Estadual de Londrina, Londrina, PR, Brasil.; ^2^ Universidade Anhanguera, Unidade Catuaí, Londrina, PR, Brasil.; ^3^ Universidade Estadual de Maringá, Maringá, PR, Brasil.

**Keywords:** Saúde Mental, Saúde Ocupacional, Local de Trabalho, Tecnologia da Informação, Software, Enfermagem

## Abstract

**Objetivo:**

desenvolver e validar o conteúdo e os aspectos técnicos de um *web software* para a promoção da saúde mental no ambiente laboral.

**Método:**

estudo metodológico aplicado e de desenvolvimento tecnológico, realizado em três etapas: 1) Desenvolvimento de *umbrella review* ; 2) Desenvolvimento de *web software* ; 3) Validação do conteúdo e técnica realizada por 14 juízes. Os dados foram submetidos à análise estatística descritiva e cálculo do índice de validade de conteúdo.

**Resultados:**

a partir das recomendações de diretrizes, foram definidas e extraídas as informações para elaborar o *web software* constituído nas seguintes dimensões: educação em saúde mental, apoio entre os colegas de trabalho, estratégias de promoção e autoavaliação da saúde mental. Para o desenvolvimento técnico, definiram-se os objetivos, as funções gerais e a infraestrutura tecnológica *.* Após o desenvolvimento e testes de funcionamento, foi disponibilizada a versão para a validação de conteúdo e técnica por juízes. O índice de validade de conteúdo global foi de 0,98 e, para os aspectos técnicos, foi de 0,97.

**Conclusão:**

a concordância entre os juízes em relação ao conteúdo e aos aspectos técnicos, bem como as sugestões incorporadas, demonstraram o potencial de uso do *web software* na promoção da saúde mental no ambiente laboral.

## Destaques

**(1)** Uso da tecnologia para fornecer informações sobre saúde mental.

**(2)** Possibilita disseminar informações de forma flexível e menos estigmatizante.

**(3)** Util para a prevenção, rastreamento e enfrentamento dos agravos à saúde mental.

**(4)** Contribui com gestores e enfermeiros do trabalho na avaliação dos trabalhadores.

**(5)** Possibilita desenvolver estratégias institucionais para promoção da saúde mental.

## Introdução

 O adoecimento mental do trabalhador tem-se configurado mundialmente como um importante problema de saúde pública e a principal causa de ausência no trabalho por doença e incapacidade ^(^ 1 ^)^ . Estima-se que 15% dos adultos apresentem algum tipo de transtorno mental no decorrer da vida laborativa. A depressão e a ansiedade têm despontado como as principais causas de incapacidade no trabalho: anualmente, ambas têm contribuído para aproximadamente 12 bilhões de dias de trabalho perdidos, custando à economia global quase um trilhão de dólares ^(^ 2 ^)^ . 

 No Brasil, entre os anos de 2007 a 2022 foram realizadas 17.681 notificações de transtornos mentais relacionadas ao trabalho ^(^ 3 ^)^ . Frente a esse cenário, têm sido discutidas as proposições legislativas para conceder incentivo fiscal às pessoas jurídicas que implantarem programas de saúde mental dentro do ambiente laboral, demonstrando um avanço importante para a saúde dos trabalhadores ^(^ 4 ^)^ . 

 No que se refere à causalidade, denota-se que o trabalho por si só não atua como causa principal no adoecimento mental. Contudo, ele pode ser elemento causal contributivo, ou seja, como provocador de distúrbio latente ou agravante de uma doença preexistente ^(^ 5 ^)^ . 

 A Organização Mundial da Saúde (OMS) e a Organização Internacional do Trabalho (OIT) fizeram um chamado às ações concretas para atender às preocupações sobre a saúde mental da população trabalhadora. Por intermédio da publicação de diretrizes, é destacado que a abordagem à saúde mental deve ser ampla e abrangente, e que é importante que os ambientes de trabalho adotem um cenário propício às mudanças, com comprometimento para combater o estigma e a discriminação, coordenando abordagens multissetoriais ^(^ 2 ^)^ . 

 A saúde mental prejudicada afeta pessoas em todo o mundo, impactando na qualidade de vida individual e econômica para os empregadores ^(^ 6 ^)^ . As evidências demonstraram que a implementação de intervenções nos locais de trabalho pode promover a saúde mental, melhorando o aspecto psicológico do indivíduo ^(^ 1 ^)^ . Fundamentando-se no presssuposto de que alguns problemas de saúde mental são evitáveis, há argumentos suficientes para que os empregadores invistam em soluções preventivas para a saúde mental dos trabalhadores ^(^ 6 ^)^ . 

 Nos últimos anos, as tecnologias digitais de saúde para *desktop* e dispositivos móveis começaram a ser utilizadas para ampliar o acesso às informações. Essas tecnologias podem aumentar a acessibilidade das estratégias de saúde mental. Também, podem reduzir os desafios da falta de profissionais de saúde, minimizando as barreiras decorrentes da necessidade do encontro presencial ^(^ 6 ^-^ 7 ^)^ . Assim, podem melhorar as lacunas de implementação de estratégias e mimetizar os problemas associados à saúde mental ^(^ 8 ^)^ . 

 Ainda, as inovações tecnológicas podem aumentar a conscientização e a adesão às medidas de prevenção, educação e apoio para os indivíduos afetados por condições de saúde mental, principalmente no ambiente de trabalho, onde as abordagens tradicionais tendem a ter baixa aceitação ^(^ 6 ^-^ 7 ^,^ 9 ^)^ . O uso da tecnologia também pode ser considerado útil e vantajoso para o acesso dos usuários, pois algumas pessoas não procurariam ajuda devido ao medo de serem estigmatizadas ^(^ 9 ^)^ . 

 O uso de tecnologias para prevenir ou melhorar os problemas de saúde mental pode incluir ações a partir de programas de *software* acessados por meio de computadores, *tablets, smartphones* , equipamentos audiovisuais, robôs e outros dispositivos. Os dispositivos possuem recursos para coletar, armazenar e recuperar informações, além de orientar os usuários na realização das atividades terapêuticas ^(^ 10 ^)^ . 

 No Brasil, a literatura evidencia que a produção de *software* tem sido direcionada para conduzir a organização dos serviços de saúde ^(^ 11 ^-^ 12 ^)^ , o gerenciamento da assistência de Enfermagem ^(^ 13 ^)^ , o ensino na Enfermagem ^(^ 14 ^-^ 15 ^)^ e o autocuidado de pacientes ^(^ 16 ^)^ . 

 Frente ao exposto e verificando-se uma lacuna na produção do conhecimento científico nacional na elaboração de tecnologias para a promoção da saúde mental dos trabalhadores, este estudo teve como pergunta de pesquisa: O *web software* desenvolvido para a promoção da saúde mental no ambiente laboral possui validade de conteúdo e técnica? 

 Para responder a esta pergunta da pesquisa, traçou-se como objetivo deste estudo desenvolver e validar o conteúdo e os aspectos técnicos de um *web software* para a promoção da saúde mental no ambiente laboral. 

## Método

### Delineamento do estudo

 Trata-se de um estudo metodológico aplicado e de desenvolvimento tecnológico, destinado a desenvolver ou refinar métodos para a obtenção, organização ou análise de dados, com foco no desenvolvimento, validação e na avaliação de ferramentas ^(^ 17 ^)^ . 

### Período

O estudo foi desenvolvido no período de janeiro de 2021 a dezembro de 2022.

### Procedimentos do estudo

 O estudo foi realizado em três etapas: 1) Para fundamentar a elaboração do *web software* foi realizado um estudo de *umbrella review* para identificar as recomendações de diretrizes para a promoção da saúde mental no ambiente laboral; 2) Desenvolvimento do *web software* ; 3) Validação de conteúdo e técnica do *web software* por juízes. 

Na primeira etapa, realizou-se a busca de revisões sistemáticas que identificaram as recomendações de diretrizes para a promoção da saúde mental no ambiente laboral.

 A busca foi realizada em cinco bases de dados: *American Psychological Association (PsycINFO), Cochrane Library,* EMBASE *National Library of Medicine National Institutes of Health* (MEDLINE via PubMed) *e Scopus* . Utilizaram-se os descritores “ *mental health”, “worpkplace”, “guidelines”, “systematic review”.* O protocolo da *umbrella review* foi registrado na plataforma Prospero CRD42023461845. 

Os resultados foram agrupados em três categorias de recomendações, sendo: a) Recomendações de prevenção primária – proteção do trabalhador; b) Recomendações de prevenção secundária – promover a saúde mental do trabalhador; e c) Recomendações de prevenção terciária – apoiar, acompanhar e reabilitar o trabalhador no retorno ao trabalho após o afastamento.

 Com base na avaliação das recomendações descritas nas diretrizes, foram definidas e extraídas as informações para os seguintes conteúdos a serem inseridos no *web software* : conscientização sobre a saúde mental, suporte social aos colegas de trabalho, estratégias de promoção e autoavaliação da saúde mental. A autoavaliação foi fundamentada na aplicação da Escala de Depressão, Ansiedade e Estresse (DASS-21), já traduzida e validada para o Brasil ^(^ 18 ^)^ . 

 Na segunda etapa, isto é, para a concepção do *web software* , foram utilizados os passos metodológicos baseados na engenharia de *software* ^(^ 19 ^)^ . Este modelo permite o desenvolvimento de um *web software* a partir de uma série de versões evolucionárias que envolvem as fases de comunicação, planejamento, modelagem, construção, avaliação, testes de funcionamento e versão para a validação. Além disso, permite as adaptações que forem necessárias ao longo da vida do *software* ^(^ 19 ^)^ . 

 Para o desenvolvimento do *web software,* realizou-se o contato com um profissional com *expertise* em desenvolvimento de sistemas por meio de reuniões sistemáticas, nas quais definiu-se o planejamento, que incluiu os principais requisitos e os recursos do *web software* , além de um cronograma preliminar para o seu desenvolvimento. 

 A modelagem do *web software* foi estabelecida por meio de fluxograma com a interface das telas. Para a construção delineou-se o *layout* visível ao usuário, com definição da disposição das telas e ícones de acesso. O *web software* foi desenvolvido baseado nos conceitos de *Responsive Web Design* , que permite que seja acessado em *desktop* , *tablet* ou *smartphone* alterando o *layout* em relação ao tamanho das telas, facilitando a visualização. A arquitetura utilizada foi a *Content Management System* (CMS). Essa ferramenta concentra diversas funcionalidades que têm como objetivo facilitar a criação e a edição do conteúdo. A linguagem de programação empregada foi *Personal Home Page* (PHP), *Hyper Text Markup Language* (HTML5), *Cascading Style Sheets* (CSS3) e *Javascript* ^(^ 20 ^)^ . 

 Para a criação do *frontend* do *web software* , que compreende a interface visível ao usuário, utilizaram-se o programa *Adobe Photoshop* ^®^ , *Javascript* com *framework* Vue.js, HTML5 e CSS3. Para o *backend* , que envolve o processamento e o armazenamento dos dados, utilizou-se o gerenciador de banco de dados MySQL. Para a gestão dos conteúdos foi utilizado um *framework* , que é gerenciado por meio do acesso ao painel administrativo da ferramenta, facilitando a atualização pelos pesquisadores. O *framework* foi hospedado no servidor Linux/Apache. Durante a fase teste, foram verificadas as necessidades de ajustes na *interface* e na funcionalidade por meio dos testes de *links* e de formulários ^(^ 20 ^)^ . 

 A terceira etapa consistiu na validação do conteúdo e dos aspectos técnicos realizada por juízes, seguindo a recomendação de seis ou mais especialistas ^(^ 21 ^)^ . A seleção dos juízes ocorreu por meio da Plataforma Lattes, por amostragem não probabilística intencional, e que atendessem aos critérios de inclusão. 

Para o juiz de conteúdo estabeleceram-se os seguintes critérios de inclusão: titulação mínima de doutor; autor/orientador de estudos relacionados à saúde do trabalhador e/ou saúde mental; no ensino, ministra/ministrou disciplinas/palestras voltadas à saúde do trabalhador e/ou saúde mental; participa/participou de grupos/projetos relacionados à saúde do trabalhador e/ou saúde mental.

 Para o juiz técnico foram estabelecidos os seguintes critérios: titulação de especialista; autor/orientador de estudos relacionados ao desenvolvimento de *software* ; no ensino, ministra/ministrou disciplinas/palestras voltadas ao desenvolvimento de *software* ; participa/participou de grupos/projetos relacionados ao desenvolvimento de *software* . 

 Foram convidados 22 juízes, mediante o contato realizado via carta-convite enviada por meio de correio eletrônico. Destes, oito não retornaram o contato, o que resultou em uma amostra de 14 juízes, sendo estes oito enfermeiros e seis especialistas em desenvolvimento de *software* . 

### Instrumentos utilizados para a coleta das informações

 Para a coleta de dados, elaboraram-se dois formulários distintos pela ferramenta do *Google Forms* . No formulário, foram inseridos o Termo de Consentimento Livre e Esclarecido (TCLE) e o endereço eletrônico para acesso ao *web software* , juntamente com os itens do instrumento de validação. Estipulou-se o prazo de 15 dias para a avaliação e o preenchimento do instrumento, e foram realizadas mais duas tentativas de reenvio com o mesmo prazo para a resposta. O não preenchimento foi compreendido como recusa. 

 O instrumento de validação de conteúdo era composto por 13 afirmativas que contemplavam itens sobre os objetivos, conteúdo, relevância e ambiente. O instrumento de validação técnica era composto por 14 afirmativas que contemplavam itens sobre a ergonomia, funcionalidade, usabilidade e ambiente. Cada uma das dimensões continha itens avaliados por meio de escala do tipo *Likert* de quatro pontos, distribuídos em não relevante, pouco relevante, relevante e altamente relevante. Ao final de cada dimensão, foi disponibilizado um espaço para sugestões dos juízes. Os instrumentos foram adaptados de estudo de validação de *web software* denominado “Aposentar-se com Saúde”, o qual foi adequado às questões para a promoção da saúde mental no ambiente laboral ^(^ 22 ^)^ . 

### Análise dos dados

 A análise dos dados obtidos foi realizada por meio de estatística descritiva, utilizando-se os valores de frequência absoluta e relativa, com auxílio dos programas *Microsoft Office Excel* ^®^ e *Statistical Package for the Social Sciences* (SPSS) ^®^ versão 25.0. Para a análise da validade técnica e de conteúdo, aplicou-se o cálculo do Índice de Validade de Conteúdo (IVC), considerando a proporção de respostas relevantes ou altamente relevantes, dividida pelo total de respostas de cada item. O IVC das dimensões do instrumento foi obtido a partir da soma dos IVCs da dimensão, dividida pelo número de itens. Por fim, para o IVC global realizou-se a soma dos IVCs individuais, dividida pelo total de itens do instrumento. Adotou-se o valor mínimo para o IVC de 0,78 ^(^ 21 ^)^ . As sugestões enviadas pelos juízes foram organizadas e analisadas conforme as dimensões do instrumento. 

### Aspectos éticos

Este estudo atende à Resolução nº 466, de 12 de dezembro de 2012, e foi previamente aprovado pelo Comitê de Ética em Pesquisa (CEP) da instituição, conforme Parecer nº 3.588.273.

## Resultados

 O *web software* foi denominado *e-LeveMente,* está hospedado em um servidor *web* e pode ser acessado por meio do *link* https://www.elevemente.com . Para o desenvolvimento, realizou-se uma reunião com os desenvolvedores, no intuito de definir os objetivos e as funções gerais, onde elaborou-se a infraestrutura tecnológica com um fluxograma de funcionamento, definindo a sequência do conteúdo, a seleção de mídias, o modo de integração das telas e suas funções, conforme a Figura 1 . 

**Figura 1 -:** Fluxograma de funcionamento do *web software* para a promoção da saúde mental no ambiente laboral. Londrina, PR, Brasil, 2023

 Para o desenvolvimento técnico definiram-se o funcionamento da tela inicial e o *layout* de apresentação ( Figura 2 ). O conteúdo foi disponibilizado no formato de texto, vídeos e imagens, no intuito de proporcionar ao usuário um ambiente convidativo e atrativo. Ao acessar o *e-LeveMente,* no canto superior direito da tela são disponibilizadas as principais seções e os recursos, que incluem: página inicial, sessão *como apoiar alguém* , sessão *promovendo sua saúde mental* e ícone *avalie sua saúde mental* ( Figura 2 ). 

A página inicial contempla as informações sobre a definição de saúde mental, os problemas comuns de saúde mental, os fatores que influenciam a saúde mental, os sinais e os sintomas de problemas de saúde mental e as consequências dos problemas de saúde mental no trabalho.

 A sessão *como apoiar alguém* fornece informações de como facilitar uma conversa inicial e abordar o assunto, o que perguntar, a escolha do local apropriado, a importância de manter a confidencialidade e tranquilizar a pessoa, como incentivar a pessoa a falar e a buscar apoio profissional, consideradas as estratégias para estabelecer uma comunicação eficaz. 

**Figura 2 -:** Representação gráfica da tela inicial e de conteúdo do *web software* para a promoção da saúde mental no ambiente laboral. Londrina, PR, Brasil, 2023

 Na seção *promovendo sua saúde* *mental* , é disponibilizado o conteúdo para o autocuidado. As estratégias incluem orientações como: importância de manter-se ativo, estabelecer um equilíbrio entre o trabalho e a vida particular, cultivar relacionamentos saudáveis, alerta sobre os riscos de fumar, beber e usar drogas para reduzir o desconforto relacionado à saúde mental, a importância de alimentar-se bem, técnicas de relaxamento, hábitos para melhorar a qualidade do sono, habilidades positivas para o enfrentamento, práticas saudáveis no local de trabalho, alerta para não se automedicar em caso de sinais e sintomas relacionados à saúde mental, práticas para a comunicação assertiva, sugestões de aplicativos para auxiliar no gerenciamento da saúde mental e as informações sobre onde procurar por ajuda profissional. 

 Em todas as seções, o ícone *avalie sua saúde mental* é disponibilizado no canto superior direito da tela. Ao selecionar *iniciar avaliação* , o usuário é direcionado para uma tela na qual visualiza o TCLE e, após leitura e concordância, é direcionado para a tela contendo o questionário de caracterização sociodemográfica e ocupacional. As perguntas na interface do *web software* são obrigatórias e possibilitam avançar para a tela seguinte, após o preenchimento completo. 

Na tela seguinte, é disponibilizado o instrumento para autoavaliação e rastreamento das condições de saúde mental. Antes de iniciar as repostas é emitida a seguinte informação: “O questionário é uma medida objetiva, confiável e clara para que o usuário entenda como está se sentindo. O resultado da avaliação não indica um diagnóstico conclusivo e sim o ponto de partida para estimular o autocuidado ou a busca por uma ajuda profissional”.

 As questões foram disponibilizadas em três telas com sete questões, de forma que o usuário insira uma opção de resposta para cada questão, e isso permite passar de uma tela para a outra de forma direta, utilizando os botões para avançar ou retornar. Ao final das respostas do questionário, o próprio *software* realiza o cálculo das pontuações de acordo com as recomendações da escala, fornecendo o escore de cada indivíduo e gerando uma página com os resultados. O resultado é entregue dividido entre os três fatores: depressão, ansiedade e estresse, e classificados em normal, mínimo, moderado, grave e muito grave. 

 De acordo com a pontuação são indicadas as estratégias para a promoção da saúde mental, assim como as possibilidades para a ajuda profissional, que são acessadas por meio do *link* disponível ( Figura 3 ). As respostas dos questionários ficam armazenadas no banco de dados do *e-LeveMente* e podem ser acessadas por meio da área administrativa e exportadas para futuras análises. 

**Figura 3:** - Representação gráfica do questionário de autoavaliação da saúde mental e da tela de resultados do web software para a promoção da saúde mental no ambiente laboral. Londrina, PR, Brasil, 2023

 Ressalta-se que, durante o desenvolvimento do *e-LeveMente* , foram realizados testes para identificar os possíveis erros na estética, conteúdo e *links* , com o intuito de construir uma ferramenta com adequada funcionalidade. Após os testes de funcionamento, foi disponibilizada a versão para a validação de conteúdo e técnica pelos juízes. 

Para validação de conteúdo obteve-se um retorno de oito juízes de conteúdo. O tempo médio de formação foi de 25,4 anos. Destes, 25% possuíam titulação de pós-doutor, 87,5% possuíam experiência em saúde do trabalhador e 87,5% possuíam experiência em saúde mental.

 No primeiro ciclo de validação de conteúdo, o IVC global foi de 0,85. Em relação às dimensões nas quais foram distribuídos os itens, o que apresentou menor IVC foi o domínio conteúdo, com IVC de 0,79. Apesar de apresentar IVC aceitável, cinco dos itens avaliados (2.2; 2.3; 2.5; 2.6 e 3.2) não atingiram o IVC mínimo adotado neste estudo. Assim, visto que os juízes realizaram sugestões, a fim de melhorar a qualidade do conteúdo do *e-LeveMente* , estas foram avaliadas para as reformulações. 

 No que concerne às considerações dos juízes, na Dimensão Objetivo foi sugerido disponibilizar as informações sobre a promoção da saúde mental de forma aberta, considerando que o usuário só teria acesso às mesmas após o preenchimento do questionário de autoavaliação. Essa sugestão foi atendida e foi criada a seção no menu principal *promovendo sua saúde mental* . 

 Na Dimensão Conteúdo, foram sugeridas as alterações no estilo da fonte e revisão gramatical das informações. No questionário de caracterização, no item sexo foi sugerido incluir a opção *prefiro não declarar* . Em relação ao questionário de autoavaliação da saúde mental, foi recomendado incluir a referência completa, e não somente o nome dos autores. 

 Nas estratégias de promoção da saúde mental, foi aconselhado substituir o termo “fomentando relacionamentos” por “cultivando relacionamentos saudáveis” e incluir a palavra “evite” antes de: fumar, beber e usar drogas. Além disso, foi sugerido incluir informações sobre a automedicação, sono e comunicação assertiva. Nas técnicas de relaxamento, a sugestão foi incluir alguns aplicativos gratuitos para *Android* e *iPhone Operating Syste* m (iOS) para o relaxamento e a meditação. 

 Para a Dimensão Relevância, foi destacado como excelente o formato utilizado para apresentar o conteúdo sobre as consequências dos problemas de saúde mental no trabalho, bem como as estratégias para ajudar o trabalhador a falar da sua saúde mental e as orientações pertinentes no *checklist* da comunicação. Também foi considerado relevante, na avaliação da sua saúde mental, orientar os resultados e as estratégias para a promoção da saúde mental. Alguns juízes reforçaram que a identificação da saúde mental estava sendo atingida, porém, as estratégias precisavam ser aprimoradas, conforme apontado na Dimensão Conteúdo. 

 Na Dimensão Ambiente, os juízes destacaram que as cores suaves e as figuras estavam adequadas e com excelente *design* . O material é de fácil abordagem, com conteúdo significativo e relevante para a saúde mental do trabalhador, vídeos bem elaborados, claros e com a capacidade de transmitir as informações e as orientações necessárias. Foi indicado, apenas, diversificar as melodias presentes nos vídeos, assim como reduzir a velocidade de reprodução, para que o usuário consiga assimilar as informações com maior tranquilidade. 

 Ressalta-se que as sugestões realizadas pelos juízes foram avaliadas e incorporadas à nova versão do *e-LeveMente* para a validação. Assim, submeteu-se a versão adequada para um novo ciclo de validação. Neste, obteve-se um retorno de sete juízes e o IVC global foi de 0,98, conforme apresentado na Tabela 1 . 

 Na validação técnica obteve-se um retorno de seis juízes. O tempo médio de formação foi de 11,8 anos, com graduação em Análise de Sistemas, Ciência da Computação, Engenharia da Computação, Engenharia de Desenvolvimento de *Software* e Rede de Computadores. Destes, 16,7% possuíam titulação de doutor, houve 33,3% com titulação de mestre e 50% eram especialistas. 

**Tabela 1 -:** Índice de Validade de Conteúdo e percentual de concordância entre os juízes de validação de conteúdo do *web software* para a promoção da saúde mental no ambiente laboral. Londrina, PR, Brasil, 2023

**Itens de validação**	**1° Percentual de Concordância**					**2° Percentual de Concordância**				
	1** %	**2** ^†^ **%**	**3** ^‡^ **%**	**4** ^§^ **%**	**IVC** ^||^	1* %	**2** ^†^ **%**	**3** ^‡^ **%**	**4** ^§^ **%**	**IVC** ^||^
**1. Objetivos** 1.1 Os objetivos do *web software* são coerentes com a promoção da saúde mental.	12,5	0,0	37,5	50,0	0,88	0,0	0,0	28,6	71,4	1,00
1.2 O *web software* facilita a compreensão da temática promoção da saúde mental.	0,0	0,0	37,5	62,5	1,00	0,0	0,0	28,6	71,4	1,00
1.3 Os objetivos propostos estão adequados à sua efetivação.	0,0	12,5	25,0	62,5	0,88	0,0	0,0	28,6	71,4	1,00
**IVC** ^||^ **- Dimensão Objetivos**					**0,92**					**1,00**
**2. Conteúdo** 2.1 Os conteúdos do *web software* correspondem aos objetivos do mesmo.	0,0	12,5	37,5	50,0	0,88	0,0	0,0	28,6	71,4	1,00
2.2 Os conteúdos do *web software* são suficientes para alcançar os objetivos propostos.	0,0	25,0	62,5	12,5	0,75	0,0	0,0	57,1	42,9	1,00
2.3 Os conteúdos do *web software* alcançam com precisão o escopo do tema.	0,0	37,5	50,0	12,5	0,63	0,0	0,0	42,9	57,1	1,00
2.4 As informações apresentadas estão corretas.	0,0	0,0	62,5	37,5	1,00	0,0	0,0	14,3	85,7	1,00
2.5 A forma de redação está adequada aos diferentes níveis de conhecimento dos trabalhadores de instituição de saúde.	0,0	25,0	25,0	50,0	0,75	0,0	0,0	28,6	71,4	1,00
2.6 Os conteúdos facilitam a compreensão das diferentes dimensões da promoção da saúde mental.	0,0	25,0	37,5	37,5	0,75	0,0	0,0	42,9	57,1	1,00
**IVC** ^||^ **- Dimensão Conteúdo**					**0,79**					**1,00**
**3. Relevância** 3.1 Os conteúdos abordam aspectos-chave que devem ser explorados na promoção da saúde mental.	0,0	12,5	25,0	62,5	0,88	0,0	0,0	14,3	85,7	1,00
3.2 Os conteúdos do *web software* são relevantes para que o trabalhador possa identificar os aspectos relacionados à saúde mental, bem como buscar estratégias para fortalecer a saúde mental.	0,0	25,0	12,5	62,5	0,75	0,0	0,0	28,6	71,4	1,00
**IVC** ^||^ **- Dimensão Relevância**					**0,82**					**1,00**
**4. Ambiente** 4.1 O ambiente é adequado para a apresentação do conteúdo.	0,0	12,5	37,5	50,0	0,88	0,0	14,3	28,6	57,1	0,86
4.2 O ambiente é adequado para a compreensão da saúde mental do trabalhador.	0,0	12,5	25,0	62,5	0,88	0,0	14,3	28,6	57,1	0,86
**IVC** ^||^ **- Dimensão Ambiente**					**0,88**					**0,86**

* 1 = Não Relevante

^†^ 2 = Pouco Relevante

^‡^ 3 = Relevante

^§^ 4 = Altamente Relevante

^||^ IVC = Índice de Validade de Conteúdo

 A validação técnica obteve IVC global de 0,97. No que se refere às dimensões do instrumento, a que apresentou menor IVC foi a Dimensão Funcionalidade, com IVC de 0,89 ( Tabela 2 ). 

 Cabe destacar ainda, que na Dimensão Funcionalidade não foi realizada uma sugestão específica do *e-LeveMente* , mas foi pontuada a melhoria na contextualização, antes de iniciar as respostas dos questionários. Para esse apontamento não foi realizada nenhuma alteração, pois considerou-se suficiente como contextualização a leitura e o aceite do TCLE, no qual o usuário, antes de responder às questões, era informado sobre os objetivos da pesquisa e no que consistiam suas respostas. Para a validação técnica foi considerado suficiente um ciclo de respostas, haja vista que não foram apontadas as alterações técnicas. 

**Tabela 2 -:** Índice de Validade de Conteúdo e percentual de concordância entre os juízes de validação técnica do *web software* para a promoção da saúde mental no ambiente laboral. Londrina, PR, Brasil, 2023

**Itens de validação**	**Percentual de Concordância**				
	1* %	**2** ^†^ **%**	**3** ^‡^ **%**	**4** ^§^ **%**	**IVC** ^||^
**1.Ergonomia** 1.1 O usuário pode se deslocar de uma tela para a outra rapidamente.	0,0	0,0	66,7	33,3	1,00
1.2 A localização dos dados é mantida de forma consistente de uma tela para a outra.	0,0	0,0	66,7	33,3	1,00
1.3 Textos e recursos de estilo (Ex.: sublinhado, negrito, itálico) são empregados adequadamente.	0,0	16,7	16,7	66,7	0,83
1.4 Controles e comandos encontram-se visualmente diferenciados das informações apresentadas nas telas.	0,0	0,0	33,3	66,7	1,00
1.5 Itens selecionados para o acionamento são destacados dos demais.	0,0	0,0	66,7	33,3	1,00
1.6 As mensagens de erro são concisas e objetivas.	0,0	0,0	50,0	50,0	1,00
**IVC** ^||^ **- Dimensão Ergonomia**					**0,97**
**2. Funcionalidade** 2.1 O *web software* está adequado para as propostas a que se destina.	0,0	0,0	16,7	83,3	1,00
2.2 O *web software* desempenha as funções propostas de forma correta.	0,0	0,0	33,3	66,7	1,00
2.3 O *web software* possibilita gerar resultados positivos.	0,0	0,0	16,7	83,3	1,00
**IVC** ^||^ **- Dimensão Funcionalidade**					**1,00**
**3. Usabilidade** 3.1 O *web* *software* é fácil de utilizar.	0,0	16,7	33,3	50,0	0,83
3.2 É fácil aprender os conceitos e as aplicações do *web* *software* .	0,0	0,0	66,7	33,3	1,00
3.3 O *web* *software* permite que o trabalhador tenha facilidade em aplicar os conteúdos abordados.	0,0	16,7	16,7	66,7	0,83
**IVC** ^||^ **- Dimensão Usabilidade**					**0,89**
**4. Ambiente** 4.1 O tempo de resposta do *web* *software* é adequado para que o trabalhador acesse os conteúdos disponibilizados nas diferentes telas.	0,0	0,0	33,3	66,7	1,00
4.2 A organização dos tópicos nas diferentes telas do *web software* é adequada para o bom entendimento do conteúdo, bem como a fácil localização do tema desejado.	0,0	0,0	33,3	66,7	1,00
**IVC** ^||^ **- Dimensão Ambiente**					**1,00**

* 1 = Não Relevante

^†^ 2 = Pouco Relevante

^‡^ 3 = Relevante

^§^ 4 = Altamente Relevante

^||^ IVC = Índice de Validade de Conteúdo

## Discussão

 O uso da tecnologia para a promoção da saúde é uma realidade em crescente desenvolvimento, principalmente após a pandemia da COVID-19, momento em que as ferramentas digitais para a gestão da saúde se difundiram exponencialmente ^(^ 23 ^)^ . 

 Na literatura é observado que enfermeiros pesquisadores têm buscado soluções tecnológicas inovadoras para a saúde. Destaca-se o desenvolvimento de *software* para acompanhamento do pré-natal ^(^ 24 ^)^ , a prevenção de lesões ^(^ 25 ^)^ , a educação do paciente no pré-operatório de revascularização miocárdica ^(^ 26 ^)^ , a avaliação do consumo alimentar ^(^ 27 ^)^ , o planejamento para a aposentadoria ^(^ 28 ^)^ e os aplicativos de apoio aos familiares e amigos próximos de pessoas deprimidas com risco potencial de autoextermínio ^(^ 29 ^)^ . 

 O *web software* desenvolvido teve como objetivo difundir o conhecimento e indicar as estratégias que colaboram para a promoção da saúde mental. No mundo do trabalho é imprescíndivel adotar iniciativas para lidar com os problemas de saúde mental. Conscientizar e melhorar a compreensão dos trabalhadores é apenas parte dos esforços para reduzir o estigma, capacitá-los a valorizar o seu bem-estar, além de reconhecer quando procurar ajuda ^(^ 30 ^)^ . 

 Revisão sistemática, que teve como objetivo identificar as evidências atuais para as intervenções com foco na redução do estigma relacionado aos problemas de saúde mental no trabalho, concluiu que a elevada proporção da força de trabalho poderia se beneficiar das intervenções destinadas a reduzir o estigma relacionado à saúde mental. Dentre essas, as intervenções *online* têm-se mostrado promissoras por serem mais curtas, além de parecerem ter os mesmos efeitos positivos das intervenções presenciais ^(^ 31 ^)^ . 

 Um estudo de revisão sistemática, que buscou caracterizar a literatura e o estado atual das plataformas *mHealth* projetadas para a ansiedade ou depressão disponíveis em lojas de aplicativos, identificou 169 e 179 aplicativos nas lojas *Google Play Store* e *Apple App Store* , respectivamente. O objetivo mais comum das plataformas, em ambas as buscas, foi o tratamento e, em apenas 12,3% dos aplicativos, foi mencionado o uso de testes validados, diretrizes de organizações internacionais, terapias validadas ou artigos revisados por pares ^(^ 32 ^)^ . 

 O advento da COVID-19 ascendeu o potencial das inovações digitais para melhorar o acesso e a qualidade do atendimento em saúde mental ^(^ 33 ^)^ . No Brasil, um estudo que relatou a implementação de um programa multimodal que ofereceu ações preventivas e o tratamento de saúde mental a 22 mil trabalhadores, demonstrou que o uso de aplicativo foi uma estratégia viável em ampla escala para a triagem da saúde mental, ofereceu informações preventivas com vídeos para o encaminhamento do atendimento profissional ^(^ 34 ^)^ . 

 Outro estudo que avaliou as intervenções baseadas em *mindfulness online* para auxiliar os profissionais de Enfermagem no autocuidado da saúde mental, evidenciou que as estratégias de educação *online* são úteis na redução do estresse percebido, dos sintomas de ansiedade e depressão, bem como propiciam a satisfação dos participantes com a vida e o trabalho ^(^ 35 ^)^ . 

 As inovações digitais na saúde mental oferecem potencial, contudo, alguns especialistas internacionais destacaram que as intervenções digitais são melhor utilizadas como um complemento para enriquecer as ações presenciais, pois o envolvimento sustentado por intervenções digitais também necessita de interação humana ^(^ 33 ^)^ . 

 No desenvolvimento do *web software e-LeveMente* , os recursos e a apresentação do conteúdo foram idealizados com o intuito de torná-lo informativo, convidativo, atrativo e de fácil usabilidade. Ressalta-se que esses aspectos têm-se destacado como desafios para o desenvolvimento de *software* ^(^ 28 ^)^ . Evidências relataram que mesmo as intervenções que são consideradas minimalistas, como o aconselhamento com acesso e apoio *online* por meio de aplicações, estão associados aos níveis mais baixos de presenteísmo ^(^ 36 ^)^ . 

 Na concepção de ferramentas tecnológicas, as características, habilidades e contexto dos usuários devem ser avaliadas ^(^ 37 ^)^ . A tomada de decisão das partes interessadas também é influenciada pela inevitabilidade de se tornar digital. Assim, fortalecer o corpo de evidências sobre as intervenções digitais é importante não apenas para informar se deve-se ou não adotar as decisões, mas, também, como se pode tornar uma prática proficiente nos cuidados de saúde mental ^(^ 10 ^)^ . 

 O cuidado digital representa um avanço na incorporação das tecnologias aos cuidados de saúde. Essa estratégia pode proporcionar ao usuário o empoderamento pelo autogerenciamento da própria saúde e fortalecer as ações preventivas e de autocuidado ^(^ 38 ^)^ . Além disso, o uso das tecnologias de informação e comunicação pode contribuir com intervenções inovadoras e oportunas, que abordem os problemas de saúde mental e promovam uma força de trabalho saudável ^(^ 39 ^)^ . 

 Um estudo que avaliou o custo-benefício das intervenções digitais em saúde mental destacou que essas estratégias podem ser preferidas, ao invés de se visitar um profissional de saúde. Além disso, a intervenção digital pode melhorar o acesso aos serviços e ao autocuidado, permitindo o cuidado contínuo ^(^ 10 ^)^ . 

 Em um estudo de revisão sistemática que avaliou 117 estudos e 11.119 participantes randomizados, identificou-se que o estresse entre os profissionais de saúde pode ser combatido em nível organizacional, mas também em nível individual. As intervenções em nível individual, como concentrar-se em pensamentos, sentimentos, comportamento, como fazer exercício, relaxar, ioga, acupuntura, podem reduzir o estresse entre os profissionais de saúde até um ano após a intervenção ^(^ 40 ^)^ . Desse modo, pode-se inferir que o *web software* proposto seja uma ferramenta acessível a ser utilizada para promover a saúde mental dos trabalhadores no âmbito singular e organizacional. 

 No que se refere à etapa de validação, constituiu-se em uma fase imprescindível, visto que possibilitou a avaliação e o aprimoramento do *web software* . Alguns estudos de validação buscaram verificar a adequação, qualidade, legitimidade e credibilidade de um objeto a ser validado com base na opinião de *experts* no tema e/ou usuários ^(^ 41 ^)^ . Além disso, o desenvolvimento dessas tecnologias tem-se constituído uma oportunidade de colaboração entre os pesquisadores da saúde e profissionais de tecnologia para projetar e desenvolver ferramentas para apoiar os profissionais de saúde e conectá-los aos usuários ^(^ 9 ^)^ . 

 Neste estudo houve a necessidade da realização de dois ciclos de revisão pelos juízes para a validação de conteúdo, mesmo obtendo um IVC global e por dimensão satisfatório. Seguiu-se a recomendação de que, se os avaliadores identificassem os aspectos do constructo não adequadamente contemplados, seria necessário fazer nova revisão ^(^ 17 ^)^ . Outro estudo, que realizou o desenvolvimento e a validação de aplicativo para acompanhamento pré-natal, também apontou a necessidade da validação em duas etapas ^(^ 24 ^)^ . 

 O IVC global técnico e de conteúdo obtido neste estudo foi considerado elevado, 0,97 e 0,98 respectivamente. A análise de validação pelo IVC é amplamente utilizada; em um estudo de revisão de escopo que avaliou 881 estudos de validação na área de Enfermagem, constatou que, em relação ao tipo de análise, a utilização do IVC ocorre em aproximadamente 40% dos estudos de validação como uma das dimensões mais importantes na análise de um material. Essa análise permite determinar a validação com base em cálculos estatísticos predefinidos ^(^ 41 ^)^ . 

 Constataram-se nas avaliações realizadas pelos juízes as diferentes percepções. Isso vai ao encontro da característica de subjetividade do método, o qual considera a individualidade da interpretação, o que pode resultar em distinções na avaliação ^(^ 41 ^)^ . 

 Na validação de conteúdo, os juízes consideraram como relevante o fato de o *web software* possibilitar a autoavaliação e classificação imediata da saúde mental em relação à depressão, ansiedade e ao estresse, e ainda sugerir ações para o autocuidado. Evidências demonstraram que as ferramentas de avaliação podem melhorar diretamente a saúde mental dos usuários, encorajando-os a refletir sobre sua própria saúde mental ^(^ 6 ^)^ . 

 A autoavaliação é uma etapa importante, pois a procura precoce por ajuda para os sintomas de saúde mental ainda é incomum, contudo, na literatura é relatado que 70% das pessoas que tinham transtorno mental clínico ou subclínico nunca haviam buscado ou recebido tratamento. Esses dados destacaram a imprescindibilidade do fornecimento de intervenções no local de trabalho para a prevenção, identificação e tratamento precoce da depressão e ansiedade em pessoas que, de outra forma, não procurariam ajuda ^(^ 1 ^)^ . 

 As tecnologias digitais também têm sido reconhecidas como essenciais para avançar nos objetivos de desenvolvimento sustentável ^(^ 7 ^)^ . Este estudo corrobora as iniciativas globais sobre a saúde digital como um meio de promover o acesso à saúde, vidas saudáveis e o bem-estar para todos, principalmente, com medidas de promoção da saúde. 

 Embora os objetivos do estudo tenham sido atingidos, o mesmo apresentou limitações relacionadas às etapas de validação do *e-LeveMente,* as quais foram restritas à avaliação de conteúdo e dos aspectos técnicos somente com juízes, não sendo realizada a validação com os trabalhadores. Assim, sugere-se que posteriormente o *web software* seja validado no ambiente laboral. Outra limitação refere-se à escassez de literatura que permitisse a comparabilidade dos resultados. 

 Ressalta-se no entanto, que o *web software* *e-LeveMente* contribui para o avanço no conhecimento científico para a promoção da saúde mental no ambiente laboral, pois oferece informações sobre a saúde mental de forma flexível, menos estigmatizante e de fácil acesso. Também poderá auxiliar gestores e enfermeiros que atuam na gestão da saúde do trabalhador, podendo ser utilizado na avaliação do perfil de saúde mental dos trabalhadores, a partir do acesso ao banco de dados e, dessa forma, possibilitar o desenvolvimento de estratégias institucionais para a promoção da saúde mental no ambiente laboral. 

## Conclusão

 O *web software* para a promoção da saúde mental no ambiente laboral *e-LeveMente* mostrou-se válido em seu conteúdo e aspectos técnicos na avaliação de juízes com *expertise* em saúde mental, saúde do trabalhador e desenvolvimento de sistemas. Considera-se que a concordância entre os juízes, bem como as sugestões incorporadas, demonstraram o potencial para o uso na promoção da saúde mental no ambiente laboral. 

 Assim, destaca-se que, embora cada local de trabalho precise desenvolver soluções personalizadas, o *web software* para a promoção da saúde mental no ambiente laboral *e-LeveMente,* após a validação com os trabalhadores, poderá se configurar em uma ferramenta capaz de contribuir para que os locais de trabalho cumpram seu papel na promoção da saúde mental.
